# Awareness and use of five imaging decision rules for musculoskeletal injuries: a systematic review

**DOI:** 10.1186/s12245-023-00555-4

**Published:** 2023-11-13

**Authors:** Priti Kharel, Joshua R. Zadro, Zhang Chen, Madii A. Himbury, Adrian C. Traeger, James Linklater, Christopher G. Maher

**Affiliations:** 1 Sydney Musculoskeletal Health, The University of Sydney, Royal Prince Alfred Hospital, Level 10 North, King George V Building, Missenden Road, PO Box M179, Sydney, NSW 2050 Australia; 2 Sydney School of Public Health, The University of Sydney, Sydney, NSW Australia; 3 Castlereagh Imaging, Sydney, NSW Australia

**Keywords:** Canadian C-Spine Rule, Canadian CT Head Rule, NEXUS guidelines, Ottawa Ankle Rules, Ottawa Knee Rules

## Abstract

**Background:**

Several validated decision rules are available for clinicians to guide the appropriate use of imaging for patients with musculoskeletal injuries, including the Canadian CT Head Rule, Canadian C-Spine Rule, National Emergency X-Radiography Utilization Study (NEXUS) guideline, Ottawa Ankle Rules and Ottawa Knee Rules. However, it is unclear to what extent clinicians are aware of the rules and are using these five rules in practice.

**Objective:**

To determine the proportion of clinicians that are aware of five imaging decision rules and the proportion that use them in practice.

**Design:**

Systematic review.

**Methods:**

This was a systematic review conducted in accordance with the ‘Preferred reporting items for systematic reviews and meta-analyses’ (PRISMA) statement. We performed searches in MEDLINE (via Ovid), CINAHL (via EBSCO), EMBASE (via Ovid), Cochrane Central Register of Controlled Trials (CENTRAL), Web of Science and Scopus databases to identify observational and experimental studies with data on the following outcomes among clinicians related to five validated imaging decision rules: awareness, use, attitudes, knowledge, and barriers and facilitators to implementation. Where possible, we pooled data using medians to summarise these outcomes.

**Results:**

We included 39 studies. Studies were conducted in 15 countries (e.g. the USA, Canada, the UK, Australasia, New Zealand) and included various clinician types (e.g. emergency physicians, emergency nurses and nurse practitioners). Among the five decision rules, clinicians’ awareness was highest for the Canadian C-Spine Rule (84%, *n* = 3 studies) and lowest for the Ottawa Knee Rules (18%, *n* = 2). Clinicians’ use was highest for NEXUS (median percentage ranging from 7 to 77%, *n* = 4) followed by Canadian C-Spine Rule (56–71%, *n* = 7 studies) and lowest for the Ottawa Knee Rules which ranged from 18 to 58% (*n* = 4).

**Conclusion:**

Our results suggest that awareness of the five imaging decision rules is low. Changing clinicians’ attitudes and knowledge towards these decision rules and addressing barriers to their implementation could increase use.

**Supplementary Information:**

The online version contains supplementary material available at 10.1186/s12245-023-00555-4.

## Introduction

A decision rule is a decision support tool designed to help clinicians provide high-quality care to patients with musculoskeletal conditions [1]. These conditions affect 1.71 billion people worldwide and are ranked as a leading cause of global disability [2]. In the USA, expenditures on healthcare related to musculoskeletal disorders reached $380.9 billion [3]. In Australia, they impose a substantial health and economic burden, surpassing costs associated with cardiovascular disease and cancer [4], particularly when accounting for indirect expenses [5]. Several validated decision rules exist to guide the appropriate use of imaging for patients with musculoskeletal injuries. These include the Canadian CT Head Rule and the Canadian C-Spine Rule, both with a sensitivity of 99–100% [6, 7]. Other rules include the National Emergency X-Radiography Utilization Study (NEXUS) guideline, the Ottawa Ankle Rules and the Ottawa Knee Rules with sensitivities of 83–100% [7], 99.4% [8] and 98.5% [9], respectively. The high sensitivity of these rules means they are useful for identifying patients who do not require diagnostic imaging because they are highly unlikely to have a serious underlying injury (e.g. fracture).

Both overuse and underuse of imaging are potential problems in the management of musculoskeletal injuries. Overuse of imaging wastes scarce healthcare resources and increases a patient’s exposure to radiation [10]. Underuse of imaging can lead to a missed diagnosis and long-term disability (e.g. due to a missed ankle fracture) [11]. Appropriate use of imaging could ensure correct diagnosis and treatment, thereby improving outcomes whilst minimising unnecessary exposure to radiation and reducing costs [12–14]. Studies have demonstrated substantial reductions in overuse of imaging [7, 9, 15] and decreased patients’ length of stay in the emergency department [16, 17] by implementing imaging decision rules. As a result, these decision rules are recommended in clinical practice guidelines [18–23] to guide the appropriate use of imaging.

Decision rules can guide the appropriate use of imaging for patients with musculoskeletal injuries; however, it is unclear to what extent clinicians are aware of the rules and are using them in practice. The primary aim of our review was to determine the proportion of clinicians that were aware of five validated imaging decision rules (Canadian CT Head Rule, Canadian C-Spine Rule, NEXUS guidelines, Ottawa Ankle Rules and Ottawa Knee Rules) [6–9] and the proportion that used them in practice. The secondary aims were to evaluate clinicians’ attitudes toward the rules and knowledge of the rules, and barriers and facilitators to adopting them.

## Methods

The systematic review was conducted in accordance with the ‘Preferred Reporting Items for Systematic Reviews and Meta-analyses’ (PRISMA) statement [24]. The review protocol was not registered because it was not within the scope of PROSPERO.

### Search strategy

MEDLINE (via Ovid), CINAHL (via EBSCO), EMBASE (via Ovid), Cochrane Central Register of Controlled Trials (CENTRAL), Web of Science and Scopus databases were searched to identify eligible studies from the earliest record to 27 September 2023. The search strategy was developed in consultation with a librarian and used a combination of keywords (Supplementary file 1). The search was conducted by one author (PK). Citation tracking was also performed for all studies found by electronic searches to identify studies missed by this process. The reference lists of included studies were hand-searched to identify studies missed by the primary electronic database search. There were no language or geographic restrictions in the search strategy, and studies in any language were eligible for inclusion. The number of studies identified by each database was recorded.

Two authors (PK and JZ) independently familiarised themselves with the inclusion/exclusion criteria ("Inclusion and exclusion criteria" section) and performed the selection of studies by sequentially screening the titles, abstracts and full texts of articles retrieved from the electronic database searches. Disagreements were resolved through discussion or consultation with a third reviewer (CM).

### Inclusion and exclusion criteria

#### Studies

Cross-sectional observational studies (surveys of practice) and retrospective audits of clinical notes were included. We also included experimental or quasi-experimental study designs (e.g. randomised controlled trials, non-randomised controlled trials, controlled before-after studies and interrupted time-series studies) that reported relevant data at baseline or in a ‘no intervention’ control group. Case series and case studies were excluded.

### Data extraction and quality assessment

Two authors (ZC and MH) independently extracted key study data from the included studies using a standardised data extraction form to record the following information: country, study design, setting, participant characteristics (health discipline, age, gender, experience), sample size, type of decision rule used and outcome data (awareness, use, attitudes, knowledge, barriers and facilitators). Discrepancies were resolved by a third reviewer (PK), who re-checked the data against the original citation. The definitions used to extract data on awareness, use, attitudes, knowledge, barriers and facilitators can be found in Table 1.

**Table 1:** Definitions of variables for data extraction

	**Variables**	**Definitions**
1.	Awareness	These data were captured through direct questions about clinicians’ awareness of the rules (e.g. Have you seen this rule before? Yes/No; If yes, how did you first become aware of the rule? Medical school/Journal articles/Continuing Medical Education (CME) course/colleague recommendation/participating in a research study/other)
2.	Use of rules	These data were captured in three different ways. Surveys of clinicians provided data on the number of clinicians who reported using the rules (e.g. Do you currently use this rule? Yes/No. If yes, how often do you apply the Ottawa Ankle Rules to patients who present with uncomplicated ankle injuries? Always/most of the time/Sometimes/Never) (#1). Audits of clinical notes provided data on the number of patients whose notes mentioned that an imaging decision rule was used to guide imaging decisions (#2) or mentioned clinical features that would indicate an imaging decision rule was used to guide imaging decisions (#3)
3.	Attitudes	These data were captured through direct questions about clinicians’ attitudes towards the rules from a list of options that they had to choose from (e.g. If you currently do not use this rule, would you consider using it in the near future? Yes/No; If no, why not?). Some questions assessed attitudes towards the decision rules by having participants state how strongly they agreed or disagreed (on a 5-point Likert scale) with the closed-ended statements about decision rules. (e.g. Oversimplified or cookbook medicine, too rigid to apply in individual patients, too time-consuming to apply in the ED, intended to cut health care costs)
4.	Knowledge	These data were captured through questions about knowledge of the rules and their components (e.g. Ottawa Knee Rule has been validated to guide decision-making for the diagnosis and detection of what clinical findings? Meniscal tears/ligament tears/fractures/ligament tears and fractures/iliotibial band syndrome) or vignettes of a clinical scenario where participants were asked to identify whether any of the patients’ signs would warrant imaging
5.	Barriers and facilitators	These data were captured through questions about the barriers and facilitators to use of the rules that included a list of options that participants had to choose from (e.g. rate how strongly you agree or disagree with the following statements about the Ottawa Ankle Rule: easy to learn/useful in my practice/easy to remember/easy to use /efficient use of my time/too much trouble to apply/too unsafe/would increase the chance of lawsuits). Some questions were open-ended asking participants to state the barriers or facilitators to using the decision rules

The methodological quality of the included studies was independently assessed by two authors (ZC and MH) using a modified version of the ‘Downs and Black’ checklist used for the assessment of the methodological quality both of randomised and non-randomised studies. We modified the 27-item Downs and Black (1998) checklist [25] and selected eight items that were relevant to our included studies: clarity in stating the objective, outcomes, characteristics of participants and findings of the study, representativeness of the sample to the source population, appropriateness of the statistical tests used and validity of the outcome measures used (Supplementary file 1). Disagreements were resolved by a third reviewer (PK or JZ).

### Data synthesis

We did not perform a formal meta-analysis on this data due to substantial variation in how outcomes were assessed and data reported. Instead, we calculated the pooled median (interquartile range, IQR) percentage of clinicians who were aware of the rules and used the rules. We pooled data on the use of rules as ‘self-reported use of the rules’, ‘documentation of use of the rules’ and ‘documentation of clinical features suggesting the rules were used’, due to differences in how each category is interpreted. Data on self-reported use of the rules was categorised as ‘most of the time/always/very often’ vs. the other options (e.g. sometimes/never) for pooling. Documentation of the use of rules and documentation of clinical features suggesting the rules were used have been defined in Table 1. Studies that reported the use of rules without the above classifications (most of the time/always/very often) were not included in the pooling. As we did not perform a formal meta-analysis, we did not weigh estimates nor calculate variance.

Pooled medians were stratified by country and clinician type (e.g. emergency physician, physiotherapist). One study reported the proportion of clinical centres where clinicians were using the Canadian C-Spine Rule. We treated these data as the proportion of clinicians [26]. We could not pool data for attitudes toward the rules, knowledge of the rules, and barriers and facilitators to adopting the decision rules as these data were too heterogeneous in terms of questions asked and response options.

### Patient and public involvement

We did not involve patients and members of the public in the design of this study.

## Results

### Study characteristics

**Fig. 1:**
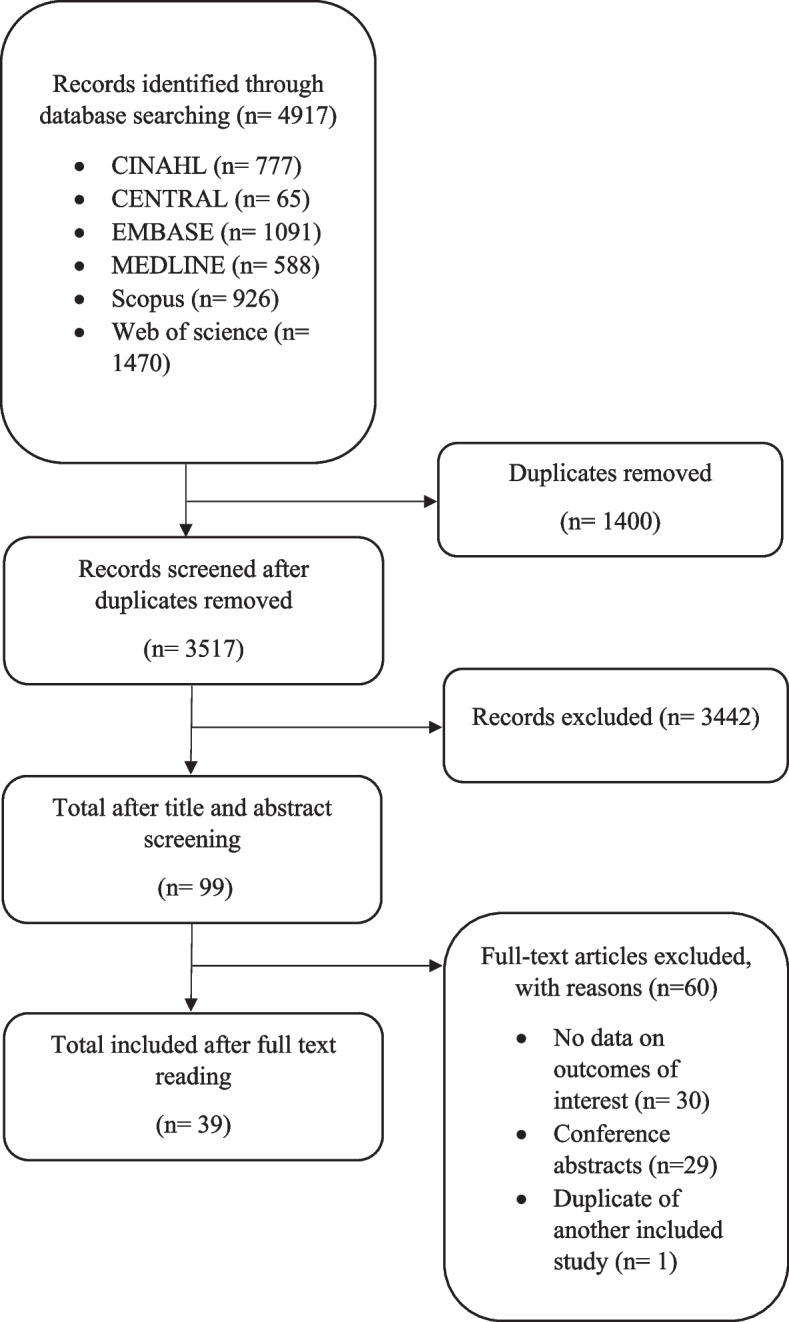
Preferred Reporting Items for Systematic Reviews and Meta-Analyses (PRISMA) flow diagram

After removing duplicates and screening 3517 titles and abstracts and 99 full-text reports, 39 studies were included (Fig. 1). Of the 39 included studies, 6 focused on the Canadian CT Head Rule [27–31], 5 on the Canadian C-Spine Rule and NEXUS combined [26, 32–35], 4 on Canadian C-Spine Rule only [36–39], 3 on NEXUS only [40–42], 18 on the Ottawa Ankle Rules [12, 43–59] and 6 on the Ottawa Knee Rules [12, 17, 49, 52, 60]. One study focused on three decision rules (Ottawa Ankle Rules, Ottawa Knee Rules, Canadian CT Head Rules) [12], and four focused on two decision rules (Ottawa Ankle Rules and Ottawa Knee Rules [12, 49, 52], Canadian C-Spine Rule and Canadian CT Head Rule [61]). The studies provided data from the USA (*n* = 11), Canada (*n* = 11), the UK (*n* = 5), Australasia (*n* = 5), New Zealand (*n* = 2) and others (*n* = 10). The study designs used were cross-sectional observational studies (*n* = 17), retrospective studies (*n* = 10), before and after study (*n* = 7) and prospective studies (*n* = 4). One study utilised both cross-sectional and retrospective data [17]. The study settings included community/tertiary/teaching hospital emergency departments (*n* = 16), major trauma centres (*n* = 7) and mixed settings due to the collection of survey data (*n* = 11). The different clinician types included emergency physicians (*n* = 22), emergency nurse practitioners (*n* = 6), physician assistants (*n* = 4), emergency nurses (*n* = 2), physiotherapists (*n* = 2), trauma team leaders (*n* = 2), junior doctors (*n* = 2) and radiologists (*n* = 1). The review included 12,048 clinicians and 7157 patients. The characteristics of the included studies are shown in (Supplementary Table 1).

### Methodological quality

Individual study scores ranged from 4 to 8 (out of a possible 8) with a mean score of 6.9 (median = 7) (Supplementary Table 2). The most common methodological limitations included participants not being representative of the population from which they were drawn (*n* = 15, 44%) and the use of outcome measures whose validity/reliability and accuracy were unknown (*n* = 7, 21%). Two studies (6%) did not clearly describe the characteristics of the participants included in the study [26, 27]. One study did not clearly describe the hypothesis/aim/objective of the study [53] and another the main findings [60]. All studies clearly described the main outcome to be measured and used appropriate statistical tests to assess the main outcomes.

### Awareness and use of imaging decision rules

The median percentage of clinicians aware of imaging decision rules was 42% (*n* = 3 studies) for the Canadian CT Head Rule, 84% (*n* = 3) for the Canadian C-Spine Rule, 71% (*n* = 5) for the Ottawa Ankle Rules and 18% (*n* = 2) for the Ottawa Knee Rules (Table 2).

**Table 2:** Awareness and use of decision rules

Total	Canadian CT Head Rule			Canadian C-Spine Rule			NEXUS			Ottawa Ankle Rules			Ottawa Knee Rule		
	**Median**	**IQR**	***N* (sample size)**	**Median**	**IQR**	***N* (sample size)**	**Median**	**IQR**	***N* (sample size)**	**Median**	**IQR**	***N* (sample size)**	**Median**	**IQR**	***N* (sample size)**
Awareness of the rules^a^	42%	31–70%	3 (2151)	84%	42–88%	3 (2016)				71%	14–73	5 (3105)	18%	5–31%	2 (1980)
Self-reported use of rules^a^	28%	25–32%	3 (2151)	56%	54–57%	2 (1559)	7%	7%	1 (1297)	57%	25–86%	5 (2817)	18%	13–23%	2 (1816)
Documentation of using the rules^b^										53%	23–55%	3 (444)			
Documentation of clinical features suggesting the rules was used^b^	53%	34–71%	2 (510)	71%	56–85%	5 (2320)	77%	59–100%	3 (3838)	59%	49–73%	6 (2316)	58%	54–63%	2 (303)

*IQR* interquartile range, *N* number of studies, *NEXUS* National Emergency X-Radiography Utilization Study guidelines

^a^The sample size denotes the patients’ sample size

^b^The sample size denotes the clinicians’ sample size

The median percentage of clinicians that use the Canadian CT Head Rule ranged from 28% (*n* = 3 studies, 2151 clinicians, assessment: self-report) to 53% (*n* = 2, 510 patients, assessment: documentation of clinical features suggesting the rules were used). The use for the Canadian C-Spine Rule ranged from 56% (*n* = 2, 1559 clinicians, assessment: self-report) to 71% (*n* = 5, 2320 patients, assessment: documentation of clinical features suggesting the rules were used). The use of NEXUS ranged from 7% (*n* = 1, 1297 clinicians, assessment: self-report) to 77% (*n* = 3, 3838 patients, assessment: documentation of clinical features suggesting the rules were used). The median percentage of clinicians that use the Ottawa Ankle Rules ranged from 53% (*n* = 3, 444 patients, assessment: documentation of using the rules) to 59% (*n* = 6, 2316 patients, assessment: documentation of clinical features suggesting the rules were used) and 18% (*n* = 2, 1816 clinicians, assessment: self-report) to 58% (*n* = 2, 303 patients, assessment: documentation of clinical features suggesting the rules were used) for the Ottawa Knee Rules (Table 2).

### Awareness and use of the decision rules by type of clinicians

#### Awareness of decision rules

The percentage of clinicians aware of the decision rules ranged from 7% (*n* = 1 study) of radiologists to 60% (*n* = 3) of emergency physicians for the Canadian CT Head Rule; 42% (*n* = 1) of the physiotherapists to 86% (*n* = 2) of emergency physicians for the Canadian C-Spine Rule; 10% (*n* = 1) of clinical educators to 87% (*n* = 2) of emergency physicians for Ottawa Ankle Rules and 5% (*n* = 1) of clinical educators to 31% (*n* = 1) in emergency physicians for Ottawa Knee Rules (Table 3).

**Table 3:** Awareness and use of the decision rules by type of clinicians

Clinicians type	Canadian CT Head Rule			Canadian C-Spine Rule			NEXUS			Ottawa Ankle Rules				Ottawa Knee Rule	
	**Median**	**IQR**	***N* (sample size)**	**Median**	**IQR**	***N* (sample size)**	**Median**	**IQR**	***N* (sample size)**	**Median**	**IQR**	***N* (sample size)**	**Median**	**IQR**	***N* (sample size)**
**Awareness of the rules**^a^															
Emergency physicians	60%	42–70%	3 (1745)	86%	84–88%	2 (1559)				87%	76–99%	2 (1964)	31%	31%	1 (1699)
Clinical educators										10%	10%	1 (211)	5%	5%	1 (211)
Family physicians										20%	20%	1 (456)			
Neurosurgeons	28%	28%	1 (179)												
Radiologists	7%	7%	1 (227)												
Physiotherapists				42%	42%	1 (457)									
**Self-reported use of rules**^a^															
Emergency physicians	32%	25–53%	3 (1745)	56%	54–57%	2 (1559)	7%	7%	1 (1297)	69%	52–86%	4 (2340)	18%	13–23%	2 (248)
Neurosurgeon	30%	30%	1 (179)												
Radiologists	4%	4%	1 (227)												
GP										3%	3%	1 (395)			
Trauma team leaders				71%	71%	1 (17)	12%	12%	1 (17)						
**Documentation of using the rules**^b^															
Emergency physicians										0	0	1 (158)			
Emergency nurse practitioners										68%	40–95%	2 (226)			
Junior doctors										70%	70%	1 (30)			
**Documentation of clinical features suggesting the rules were used**^b^															
Emergency physicians	52%	34–71%	2 (510)	70%	49–91%	2 (1041)	97%	68–100%	3 (1614)	77%	56–82%	3 (623)	58%	54–63%	2 (303)
Emergency nurse practitioners										71%	71%	1 (51)			
Physiotherapists				79%	79%	1 (457)									

*GP* general practitioners, *IQR* interquartile range, *N* number of studies, *NEXUS*, National Emergency X-Radiography Utilization Study guidelines

^a^The sample size denotes the patients’ sample size

^b^The sample size denotes the clinicians’ sample size

#### Use of decision rules

The median percentage of emergency physicians that use imaging decision rules ranged from 32% (*n* = 3 studies, assessment: self-report) to 52% (*n* = 2, assessment: documentation of clinical features suggesting the rules were used) for the Canadian CT Head Rule, 56% (*n* = 2, assessment: self-report) to 70% (*n* = 2, assessment: documentation of clinical features suggesting the rules were used) for the Canadian C-Spine Rule, 7% (*n* = 1, assessment: self-report) to 97% (*n* = 3, assessment: documentation of clinical features suggesting the rules were used) for the NEXUS, 0% (*n* = 1, assessment: documentation of using the rules) to 77% (*n* = 3, assessment: documentation of clinical features suggesting the rules were used) for the Ottawa Ankle Rules and 18% (*n* = 2, assessment: self-report) to 58% (*n* = 2, assessment: documentation of clinical features suggesting the rules were used) for the Ottawa Knee Rules (Table 3). The median percentage of emergency nurse practitioners that use the Ottawa Ankle Rules ranged from 68% (*n* = 2 studies, assessment: documentation of using the rules) to 71% (*n* = 1, assessment: documentation of clinical features suggesting the rules were used). The median percentage of neurosurgeons and radiologists that use the Canadian CT Head Rule, assessment: self-report, was 30% (*n* = 1) and 4% (*n* = 1), respectively (Table 3).

### Awareness and use of the decision rules stratified by country

#### Awareness of decision rules

The median percentage of clinicians aware of the decision rules ranged from 31% (*n* = 1 study) in the USA and Turkey to 86% (*n* = 1) in Canada for the Canadian CT Head Rule; 65% (*n* = 1) in the USA to 94% (*n* = 1) in Australasia for the Canadian C-Spine Rule; 10% (*n* = 1) in Australia to 99% (*n* = 3) in Canada for Ottawa Ankle Rules and 5% (*n* = 1) in Australia to 63% (*n* = 1) in Canada for Ottawa Knee Rules (Table 4).

**Table 4:** Awareness and use of the decision rules by country

**Countries**	**Canadian CT Head Rule**			**Canadian C-Spine Rule**			**NEXUS**			**Ottawa Ankle Rules**			**Ottawa Knee Rule**		
	**Median**	**IQR**	***N* (sample size)**	**Median**	**IQR**	***N* (sample size)**	**Median**	**IQR**	***N* (sample size)**	**Median**	**IQR**	***N* (sample size)**	**Median**	**IQR**	***N* (sample size)**
**Awareness of the rules**^a^															
Australasia	82%	82%	1 (417)	94%	94%	1 (417)									
Australia										10%	10%	1 (211)	5%	5%	1 (211)
Canada	86%	86%	1 (339)	84%	42–97%	3 (1058)				99%	71–100%	3 (1044)	63%	63%	1 (369)
China	42%	42%	1 (247)												
France										69%	69%	1 (535)	12%	12%	1 (536)
Spain										21%	21%	1 (270)	9%	9%	1 (271)
Turkey	31%	31%	1 (607)							20%	20%	1 (456)			
UK	66%	66%	1 (302)	90%	90%	1 (302)				91%	91%	1 (295)	29%	29%	1 (297)
USA	31%	31%	1 (239)	65%	65%	1 (239)				96%	96%	1 (227)	53%	53%	1 (120)
**Self-reported use of rules**^a^															
Australasia	32%	32%	(417)	47%	47%	(417)	16%	16%	(417)						
Canada	57%	57%	1 (339)	57%	14–73%	3 (756)	13%	13%	1 (155)	82%	56–90%	3 (1013)	17%	17%	1 (369)
China	25%	25%	1 (247)												
France										31%	31%	1 (535)	3%	3%	1 (536)
New Zealand										3%	3%	1 (395)			
Spain										9%	9%	1 (270)	4%	4%	1 (271)
Turkey	28%	28%	1 (607)												
UK	21%	21%	1 (302)	67%	63–71%	2 (319)	12%	12%	1 (17)	73%	73%	1 (295)	10%	10%	1 (297)
USA	12%	12%	1 (239)	30%	30%	1 (239)				31%	31%	1 (227)	31%	23–39%	2 (313)
**Documentation of using the rules**^b^															
UK										54%	53–55%	2 (414)			
USA													23%	23%	1 (30)
**Documentation of clinical features suggesting the rules were used**^b^															
Australia				62%	62%	1 (406)				62%	54–79%	3 (1091)			
Belgium				91%	91%	1 (281)	97%	97%	1 (281)						
Canada				79%	79%	1 (457)									
Ireland													54%	54%	1 (43)
Malta										77%	77%	1 (90)			
Netherlands							100%	100%	1 (573)						
Singapore	71%	71%	1 (349)												
Sweden	34%	34%	1 (161)												
USA				60%	49–71%	2 (1176)	73%	64–80%	4 (2984)	46%	36–56%	2 (1158)	63%	63%	1 (260)

*IQR* interquartile range, *N* number of studies, *NEXUS* National Emergency X-Radiography Utilization Study guidelines, *UK* United Kingdom; *USA* United States of America

^a^The sample size denotes the patients’ sample size

^b^The sample size denotes the clinicians’ sample size

#### Use of decision rules

The percentage of clinicians that use the decision rules ranged from 12% (*n* = 1 study, assessment: self-report) in the USA to 71% (*n* = 1, assessment: documentation of clinical features suggesting the rules were used) in Singapore for the Canadian CT Head Rule; 30% (*n* = 1 study, assessment: self-report) in the USA to 91% (*n* = 1, assessment: documentation of clinical features suggesting the rules were used) in Belgium for the Canadian C-Spine Rule; 12% (*n* = 1 study, assessment: self-report) in the UK to 100% (*n* = 1, assessment: documentation of clinical features suggesting the rules were used) in the Netherlands for the NEXUS; 3% (*n* = 1 study, assessment: self-report) in New Zealand to 82% (*n* = 3, assessment: self-report) in Canada for the Ottawa Ankle Rules and 3% (*n* = 1 study, assessment: self-report) in France to 63% (*n* = 1, assessment: documentation of clinical features suggesting the rules were used) in the USA for the Ottawa Knee Rules (Table 4).

### Attitude towards decision rules

#### Clinical decision rules

Graham (1998) found only 43% of physicians believed clinical decision rules protect against complaints, and 77% believed they are intended to cut healthcare costs [49]. Physicians in the study disagreed/strongly disagreed that decision rules were too time-consuming to apply in the emergency department (ED) (90%) and too rigid to apply to individual patients (73%)^49^ (Supplementary Table 3).

#### Canadian CT Head Rule

Four studies assessed physicians’ attitudes towards the use of the Canadian CT Head Rule [27, 31, 49, 61]. Most emergency physicians who reported not currently using the rule considered using it in the future (63% [61], 68% [27]), while many others agreed to adopt the rule if it was being used by colleagues who were happy with it (50%) [31]. Graham (1998) reported that some emergency physicians were only willing to use a rule that was 100% sensitive (52%) [49].

#### Canadian C-Spine Rule/NEXUS

Five studies assessed clinicians’ self-reported attitudes towards the Canadian C-Spine Rule/NEXUS [12, 33, 36, 38, 61]. The proportion of clinicians not currently using these rules, but considering using them, ranged from 61 [61] to 97% [38]. Two studies reported that some emergency physicians had a negative attitude towards using these rules, where 14 [33]–20% [36] responded that they would not consider using them in the future. Brehaut (2006) found that most emergency physicians considered the Canadian C-Spine Rule useful in their practice (88%), easy to use (76%), easy to learn (74%) and easy to remember (60%) [36].

#### Ottawa ankle rules

Two studies measured self-reported attitudes towards the Ottawa Ankle Rules [47, 48]. Brehaut (2005) found that most emergency physicians considered the Ottawa Ankle Rules easy to learn (96%), easy to use (95%), useful in their practice (93%) and easy to remember (89%) [47]. Clinicians from Cameron (1999) reported that they were confident the rules were supported by evidence-based research (82%) and were likely or very likely to use the rules in their clinical setting (69%) [48].

#### Ottawa knee rules

Graham (1998) assessed physicians’ attitudes towards using the Ottawa Knee Rules and found that 84% were willing to use the rule [49].

### Knowledge about the decision rules

#### Canadian CT Head Rule

Two studies assessed knowledge of the Canadian CT Head Rule [28, 31]. Ozan (2018) found that 31% of clinicians (emergency physicians, neurosurgeons and radiologists) rate their knowledge of the Canadian CT Head Rule to be sufficient, 33% rate it as insufficient and 36% rate it as absent [28]. Zakhari (2016) assessed clinicians’ content knowledge of the Canadian CT Head Rule using questions based on four clinical scenarios and found knowledge scores varied by clinician type (attending physicians − 59% correct answers, nurse practitioners − 51%, physician assistants − 46%, postgraduate year 1 − 75%, postgraduate year 2 − 50%, postgraduate year 3 − 25%, registered nurses − 44%) [31] (Supplementary Table 3).

#### Ottawa ankle rules

One study assessed medical students’ and residents’ knowledge of the Ottawa Ankle Rules [50] on a visual analogue scale (0–100; higher scores reflect greater knowledge). Mean knowledge scores ranged from 27 to 43.

#### Ottawa knee rules

Beutel (2012) assessed physicians’ knowledge of the Ottawa Knee Rules using three vignettes and two guideline questions. Only 2% answered all questions correctly, and 79% answered all but 1 of the questions correctly [17].

### Barriers and facilitators to using decision rules

The barriers and facilitators for using the rules varied across studies. Emergency physicians in Graham (2001) reported barriers such as clinical decision rules being too simplistic (15%) and rigid (13%), increased likelihood of being sued (17%), and being difficult (6%) and time-consuming (7%) to use [12] (Supplementary Table 3). Clinician-reported barriers to adopting the Canadian C-Spine Rule/NEXUS were lack of research to support their use (64%) [33], the rules being too complicated (63%) [36], not being aware of guidelines produced at their centres (50%) [33], lack of time at triage to use the rules/ED department being too busy (39%), heavy workload making it difficult to apply the rules (37%) [37, 62] and physicians not being on-board (13.2%) [62]. Facilitators included having a laminated flowchart in the office (89%), online access to the rule (56%), teamwork between nurses/physicians/management (46.9%) [62], video depiction of the rule during patient simulation (41%) [38] and reminders/emails/signs (24.8%) [62] (Supplementary Table 3).

## Discussion

This is the first systematic review to investigate the use and awareness of validated imaging decision rules (Canadian CT Head Rule, Canadian C-Spine Rule, NEXUS guidelines, Ottawa Ankle Rules and Ottawa Knee Rules) among clinicians. Among the five decision rules, clinicians were most aware of the Canadian C-Spine Rule (84%, *n* = 3 studies) followed by the Ottawa Ankle Rules (71%, *n* = 5), the Canadian CT Head Rule (42%, *n* = 3) and the Ottawa Knee Rules (18%, *n* = 2). The most used rule was the NEXUS guidelines (median percentage ranging from 7 to 77%, *n* = 4), followed by the Canadian C-Spine Rule (56 to 71%, *n* = 7 studies), Ottawa Ankle Rules (53 to 59%, *n* = 11), Ottawa Knee Rules (18 to 58%, *n* = 4) and Canadian CT Head Rule (28 to 53%, *n* = 5). Most clinicians have positive attitudes towards the decision rules or would consider using them in the future if they were not already. In terms of knowledge, studies showed consistent results with the majority showing poor knowledge of the rules among clinicians and only a quarter of clinicians having sufficient knowledge of the rules. Our systematic review highlights that there is sufficient room to raise awareness of these decision rules and promote their use among clinicians who manage people with acute musculoskeletal injuries.

### Strengths and weaknesses of the study

Strengths of this review include using a comprehensive search strategy to identify studies on clinicians’ awareness of, use of, knowledge of and attitudes towards five validated imaging decision rules (Supplementary file 1), a large sample size (*n* = 12,048 clinicians and 7157 patients) and using two researchers to independently extract data from the included studies (further checked by a third researcher) to ensure accuracy. Limitations include most of the included studies being conducted in developed countries (e.g. the UK, the USA, Canada, Australia, France, Spain), and variation in how knowledge about the rules was assessed across studies. Thus, the results may not be generalisable to developing countries. Another limitation was that samples in the included studies were not randomly drawn from the population of interest and may not have been representative.

### Meaning of the study

Our review highlighted that many clinicians are not aware of imaging decision rules, and among those who are aware, many do not use them despite some of them being validated more than 25 years ago [63, 64]. Clinicians’ use of these rules could be directly or indirectly affected by their knowledge and attitude towards them. For example, only one-third of the clinicians in a study indicated that they have sufficient knowledge about the Canadian CT Head Rule [28]. Regarding attitudes, some clinicians do not use the rules and do not plan on using them in the future [33, 36], while others would consider using them in the future [38, 61], particularly if colleagues start using them [31]. Clinicians also reported facilitators to use the decision rules. Facilitators of using the Canadian C-Spine Rule include beliefs that the decision rules are easy to learn, use and remember and useful in their practice [37]. Other facilitators include having a poster of the rules in the workplace, being eager to take on new responsibilities, and involvement in research projects [37].

Across the five validated decision rules investigated, clinicians are most aware of the Canadian C-Spine Rule (84%) and its use is second highest among clinicians (56–71%) after the NEXUS guidelines (7–77%). This could be because the majority of the included studies were conducted in Canada, where the rules were developed and validated first and where usage was the highest (Supplementary Table 1) [65, 66]. Emergency physicians’ awareness, use and knowledge of the rules appeared to be higher than that for other clinicians (clinical educators, neurosurgeons, radiologists, general practitioners, junior doctors and physiotherapists). For example, 86% of emergency physicians’ were aware of the Canadian C-Spine Rule compared to 42% of physiotherapists. Similarly, 97% of emergency physicians used the NEXUS guideline compared to 12% of trauma team leaders. For knowledge, the emergency physicians’ knowledge (60% had sufficient knowledge) about the Canadian CT Head Rule was higher than neurosurgeons (28%) and radiologists (8%) (Table 3).

The variability in the use of some of the decision rules was also interesting, with use ranging from 7 to 77% for the NEXUS guideline and 18–58% for the Ottawa Knee Rules. Variations in the aims of the studies, the year the studies were conducted, the countries the studies were conducted in and the assessment methods used to measure the use of the decision rules might explain some of this variation. For example, one study found only 7% of clinicians use the NEXUS guideline, which may be due to the fact that the study was a survey of emergency physicians from multiple countries (Australasia, Canada, the UK and the USA) with the main aim of collecting the data on awareness and use of Canadian C-Spine Rule and Canadian CT Head Rule (i.e. not the NEXUS guidelines specifically). While 77% of median use is from three countries where the majority of the participants were emergency physicians. As emergency physicians are one of the first points of contact for patients in a hospital emergency department, it is very important for them to be aware of validated imaging decision rules as their assessment determines whether a patient would require radiography.

### Comparison to existing research

Reported barriers for not using the decision rules included difficulty in remembering the criteria of the decision rules [65], the rules being complicated [36], patient expectation and satisfaction [17], lack of time or heavy workload [37], fear of malpractice [27] or lawsuits [37], and perceived lack of research to support their use [61]. Similar barriers were also reported in a systematic review (*n* = 76 studies) that assessed barriers to clinicians’ adherence to clinical practice guidelines. They found barriers including guidelines being inconvenient and difficult to use, resistance from patients in using guideline-based care, being short-staffed, lack of reminder systems and increased fear of liability [67].

### Unanswered questions and future research

Our systematic review highlights the need for efforts to increase the awareness and use of validated imaging decision rules among clinicians. Studies have investigated various strategies to increase the uptake of these decision rules among clinicians, with promising results. For example, one study showed that strategies such as the use of meetings, posters and pocket cards helped reduce radiography requests in the emergency department and that a minimal post-intervention implementation strategy using posters alone was effective at sustaining the intervention effect [68]. Another study showed that providing specific radiography request forms, reminders, audit and feedback and use of radiographers as ‘gatekeepers’ for imaging requests increased documentation of the Ottawa Ankle Rules and reduced imaging rates [45]. Despite several studies investigating strategies to increase the use of imaging decision rules, there has yet to be a synthesis of this available evidence. A systematic review on this topic would help clinicians understand the most effective implementation strategy and the magnitude of the effect of various strategies. This synthesis would be valuable for clinicians to determine the most effective implementation strategy to enhance the utilisation of imaging decision rules. Our review also found that most of the included studies were conducted in developed countries. Hence, future research could investigate clinicians’ awareness and use of these decision rules in developing countries, to understand the specific needs and challenges faced by clinicians treating musculoskeletal injuries in those regions.

## Conclusion

Our results suggest that there is sufficient room to raise awareness of the five decision rules and promote their use among clinicians who manage people with acute musculoskeletal injuries. Changing clinicians’ attitudes and knowledge towards these decision rules and addressing barriers to their implementation may be a necessary first step to increasing the use of these rules.

### Supplementary Information

**Additional file 1:** Search strategy. **Table S1.** Characteristics of all the included studies. **Table S2.** Methodological quality ratings of included studies using a modified ‘Downs and Black’ checklist. **Table S3.** Summary of results from included studies.

## Data Availability

All data generated or analysed during this study are included in this published article (and its supplementary information files).
