# Supplementary material for: Awareness and use of five imaging decision rules for musculoskeletal injuries: a systematic review
Source: Int J Emerg Med. 2023 Nov 13;16:85. doi: 10.1186/s12245-023-00555-4 (PMC10644430; doi:10.1186/s12245-023-00555-4)
Supplement: Supplementary file 1 — Additional file 1: Search strategy. Table S1. Characteristics of all the included studies. Table S2. Methodological quality ratings of included studies using a modified ‘Downs and Black’ checklist. Table S3. Summary of results from included studies. [file 12245_2023_555_MOESM1_ESM.doc]

**Supplementary File 1: Search strategy**

**Ovid MEDLINE(R) ALL 1946 to 25th Sept 2023**

1. Ott#wa adj3 rule*.mp
2. Ankle rule*.mp
3. Ott#wa ankle guideline*.mp
4. Ott#wa foot and ankle rule*.mp
5. Knee rule*.mp
6. (Canadian adj3 rule*).mp
7. C-spine rule*.mp
8. Cervical-spine rule*.mp
9. Spine rule*.mp
10. nexus guideline*.mp
11. national emergency x-radiography utili#ation stud*.mp
12. (nexus adj3 criteria*).mp
13. ("nexus" and "guideline*").mp
14. Canadian Computed Tomography head rule*.mp.
15. Head rule*.mp
16. 1 or 2 or 3 or 4 or 5 or 6 or 7 or 8 or 9 or 10 or 11 or 12 or 13 or 14 or 15
17. limit 16 to humans

**Embase Classic 1947 to 1973 and Embase 1974 to 2023 September 25**

1. (Ott#wa adj3 rule*).mp.
2. Ankle rule*.mp.
3. (Ott#wa adj3 guideline*).mp.
4. (Ott#wa foot and ankle rule*).mp. [mp=title, abstract, heading word, drug trade name, original title, device manufacturer, drug manufacturer, device trade name, keyword, floating subheading word, candidate term word]
5. Knee rule*.mp.
6. (Canadian adj3 rule*).mp.
7. C-spine rule*.mp.
8. Cervical-spine rule*.mp.
9. Spine rule*.mp.
10. nexus guideline*.mp.
11. national emergency x-radiography utili#ation stud*.mp.
12. (nexus adj3 criteria*).mp.
13. ("nexus" and "guideline*").mp.
14. Canadian Computed Tomography head rule*.mp.
15. Head rule*.mp.
16. 1 or 2 or 3 or 4 or 5 or 6 or 7 or 8 or 9 or 10 or 11 or 12 or 13 or 14 or 15
17. limit 16 to humans

**Cochrane Central Register of Controlled Trials August 2023**

1. (Ott#wa adj3 rule*).mp.
2. Ankle rule*.mp.
3. (Ott#wa adj3 guideline*).mp.
4. (Ott#wa foot and ankle rule*).mp. [mp=title, original title, abstract, mesh headings, heading words, keyword]
5. Knee rule*.mp.
6. (Canadian adj3 rule*).mp.
7. C-spine rule*.mp.
8. Cervical-spine rule*.mp.
9. Spine rule*.mp.
10. national emergency x-radiography utili#ation stud*.mp.
11. (nexus adj3 criteria*).mp.
12. ("nexus" and "guideline*").mp.
13. Head rule*.mp.
14. 1 or 2 or 3 or 4 or 5 or 6 or 7 or 8 or 9 or 10 or 11 or 12 or 13

**Scopus search September 2023**

1. Ott?wa w/3 rule*
2. “Ankle rule*”
3. “Ott?wa ankle guideline*”
4. “Ott?wa foot and ankle rule*”
5. “Knee rule*”
6. Canadian w/3 rule*
7. “C-spine rule*”
8. “Cervical-spine rule*”
9. “Spine rule*”
10. “nexus guideline*”
11. “national emergency x-radiography utili?ation stud*”
12. nexus w/3 criteria*
13. "nexus" and "guideline*"
14. “Canadian Computed Tomography head rule*”
15. “Head rule*”

( TITLE-ABS-KEY ( ott?wa W/3 rule* ) ) OR ( TITLE-ABS-KEY ( "Ankle rule*" ) ) OR ( TITLE-ABS-KEY ( "Ott?wa ankle guideline*" ) ) OR ( TITLE-ABS-KEY ( "Ott?wa foot and ankle rule*" ) ) OR ( TITLE-ABS-KEY ( "Knee rule*" ) ) OR ( TITLE-ABS-KEY ( canadian W/3 rule* ) ) OR ( TITLE-ABS-KEY ( "C-spine rule*" ) ) OR ( TITLE-ABS-KEY ( "Cervical-spine rule*" ) ) OR ( TITLE-ABS-KEY ( "Spine rule*" ) ) OR ( TITLE-ABS-KEY ( "nexus guideline*" ) ) OR ( TITLE-ABS-KEY ( "national emergency x-radiography utili?ation stud*" ) ) OR ( TITLE-ABS-KEY ( nexus W/3 criteria* ) ) OR ( TITLE-ABS-KEY ( nexus W/3 criteria* "nexus" AND "guideline*" ) ) OR ( TITLE-ABS-KEY ( "Canadian Computed Tomography head rule*" ) ) OR ( TITLE-ABS-KEY ( "Head rule*" ) )

**CINAHL via EBSCO September 27, 2023**

- 1. S1 "“Ott?wa w3 rule*”"
  2. S2 "“Ankle rule*”"
  3. S3 "“Ott?wa ankle guideline*”"
  4. S4 "“Ott?wa foot and ankle rule*”"
  5. S5 "“Knee rule*”"
  6. S6 ""Canadian w3 rule*""
  7. S7 "“C-spine rule*”"
  8. S8 "“Cervical-spine rule*”"
  9. S9 "“Spine rule*”"
  10. S10 "“nexus guideline*”"
  11. S11 "“national emergency x-radiography utili?ation stud*”"
  12. S12 "“nexus w3 criteria*”"
  13. S13 ""nexus" and "guideline*""
  14. S14 "“Canadian Computed Tomography head rule*”"
  15. S15 "“Head rule*”"
  16. S16 S1 OR S2 OR S3 OR S4 OR S5 OR S6 OR S7 OR S8 OR S9 OR S10 OR S11 OR S12 OR S13 OR S14 OR S15

**Web of Science Core Collection (September 27, 2023)**

1. (Ott?wa ankle rule*)
2. ("Ott?wa rule*")
3. (Ott?wa ankle guideline*)
4. (Ott?wa foot and ankle rule*)
5. ("knee rule*")
6. ("Ottawa knee rule*")
7. C-spine rule*
8. "C-spine rule*"
9. "Cervical-spine rule*"
10. “Spine rule*”
11. “nexus guideline*”
12. “national emergency x-radiography utili?ation stud*”
13. "nexus criteria*"
14. “Canadian Computed Tomography head rule*”
15. “Canadian CT head rule*”
16. “head rule*”

**Supplementary Table 1: Characteristics of all the included studies.**

| S. No. | Author (Year), country, study design |  | Study setting | Clinician included | Patients included | Demographics | Decision rule |
| --- | --- | --- | --- | --- | --- | --- | --- |
|  | Allerston (2000)  **Country:** UK  **Study design:** Retrospective observational study |  | One A&E department in Lincoln | - Emergency physicians  - Emergency nurse practitioners  Sample size unclear | 354 adults (aged 18 years or older) presenting with ankle trauma (less than 10 days post-injury) | Not reported | Ottawa Ankle Rules |
|  | Ashurst (2014)  **Country:**  USA  **Study design:**  Before and after study |  | One tertiary care centre & one urban ED in Bethlehem, Pennsylvania | - Physician assistants - Emergency nurse practitioners - Medical staff - Junior doctors - Triage nurses - Attending physicians   Sample size unclear | 30 adults (aged 18 years or older) presenting with an isolated traumatic ankle or foot injury with no other complaints or injuries present | **Patients**  **Pre-intervention period,** **n=30**   - Mean age (SD): n=36.5 (16.58) years* - Females: n=37 (61.7%)   **Clinicians**  Not reported | Ottawa Ankle Rules |
|  | Belot (2017)  **Country:**  Canada  **Study design:**  Cross-sectional observational study |  | Survey across British Columbia | 457 orthopaedic physiotherapists | N/A | **Clinicians**   - Practicing >5 years: n=339 (72.6%) - Practiced in private clinics: n=421 (92.3%). - Practised in orthopaedic or sports physiotherapy: n=445 (97.4%) | Canadian C-Spine Rules |
|  | Benayoun (2016)  **Country:**  USA  **Study design:**  Retrospective cross-sectional study |  | One Level I trauma center in Atlanta | Emergency physicians  Sample size unclear | 760 adults (16 years and older) presenting to ED following a fall and undergoing a C-Spine CT | **Patients**   - Mean age (range): 53.6 (16-99) years - Older adults (65+yrs) n=208 - Females: n=277 (36.5%)   **Clinicians**  Not reported | NEXUS & Canadian C-Spine Rules |
|  | Bessen (2009)  **Country:**  Australia  **Study design:**  Before and after study |  | EDs of a tertiary teaching hospital and a community hospital in Adelaide | 377 Emergency physicians   - Triage nurses - Nurse practitioners - Medical staff | 459 adults presenting with an ankle injury | **Patients**  **Pre-intervention period, n=459**   - Tertiary: n=215 (46.8%) - Community: n=244 (53.2%)   **Clinicians**   - Tertiary: n=315 (83.6%) - Community: n=62 (16.4%) | Ottawa Ankle Rules |
|  | Beutel (2012)  **Country:**  USA  **Study design:**  Cross-sectional & retrospective observational study |  | Three affiliated academic EDs in Providence | 47 emergency physicians | 260 patients aged 13 years or older presenting with a knee injury | **Patients**   - Mean age (median, IQR): 43.3 (41.5, 39) - Females: n= 127 (48.5%) - Insurance coverage: n=209 (80.4%) - Previous ipsilateral knee injury: n=19 (7.3%)   **Injury setting: n=235 (90%)**   - Home: n=109 (41.9%) - Street: n=64 (24.6%) - Sports: n=36 (13.8%) - Other: n=26 (10.0%)   **Mechanism of injury: n=224 (86%)**   - Fall: n=157 (60.4%) - Twisting: n=38 (14.6%) - Direct blow: n=29 (11.2%)   **Clinicians**   - Mean (SD) work hours per week in ED: 22.3 (7.8) - Mean (SD) years of experience: 8.7 (8.3) years | Ottawa Knee Rule |
|  | Borg (2008)  **Country:**  Malta  **Study design:**  Prospective observational study |  | One A&E Department in [Pietà](https://en.wikipedia.org/wiki/Piet%C3%A0,_Malta) | Accident & Emergency physicians  Sample size unclear | 73 patients aged 16 years or older presenting with an isolated ankle/midfoot injury | Not reported | Ottawa Ankle Rules |
|  | Brehaut (2005)  **Country:**  Canada  **Study design:**  Cross-sectional observational study |  | Survey of emergency physicians | 262 emergency physicians | N/A | **Clinicians**   - Mean age (SD, range): 40.9 (7.5, 28-63) - Females: n= 63 (24%) - Mean (SD, range) years since graduation: 14.5 (67.7, 1–35) years   **Work status**   - Full-time: n=190 (72.5%) - Part-time: n=61 (23.3%) - Resident: n=11 (4.2%) - Mean (SD) time per week devoted to patient care: 27.0 (11.4) hours   **Hospital setting**   - Teaching hospital: n=105 (40.0%) - Community Hospital (teaching): n=69 (26.2%) - Community Hospital (non-teaching): n=89 (33.8%) | Ottawa Ankle Rules |
|  | Brehaut (2006)  **Country:**  Canada  **Study design:**  Cross-sectional observational study |  | Survey among Canadian emergency physicians | 262 emergency physicians | N/A | **Clinicians**   - Females: n= 63 (24%) - Had emergency speciality credentials: n=216 (82.6%.) - Worked full-time in the emergency department: n=190 (72.5%) - Worked in teaching hospitals: n=174 (66.3%) - Mean hours worked per week: 27   Mean age (SD):   - Total sample (n=262): 41.63 (no SD reported) years - Never seen C-Spine Rule (n = 43): 44.4 (8.2) years - Seen but do not use (n =54): 40.4 (7.8) years - Use C-Spine Rule (n=165): 40.1 (7.0) years | Canadian C-Spine Rules |
|  | Burns (2011)  **Country:**  Canada  **Study design:**  Cross-sectional observational study |  | Tertiary care hospitals in Halifax | 155 clinicians   - Canadian pediatric emergency physicians - Trauma team leaders | N/A | **Clinicians**  **Age**   - 25–34 years: n=24 (15.5%) - 35–44 years: n=83 (53.5%) - 45–54 years: n=40 (25.2%) - 55 years: n=9 (5.8%)   **Physician training**   - General paediatrics with emergency fellowship: n=64 (41.3%) - General paediatrics: n=37 (23.9%) - Emergency medicine: n=16 (10.3%) - Paediatric critical care: n=15 (9.7%) - Other: n=19 (12.3%) - Unknown: n=4 (2.6%)   **Years in practice**   - <5 years: n=38 (24.5%) - 5–10 years: n=44 (28.4%) - >10 years: n=73 (47.1%)   **Trauma team leader**   - Yes: n=68 (43.9%) - No: n=83 (53.5%) - Unknown: n=4 (2.6%)   **Number of paediatric C-spines cleared in the last year**   - 1–9: n=29 (18.7%) - 10–19: n=35 (22.6%) - 20–29: n=22 (14.2%) - ≥30: n=70 (44.5%) | NEXUS & Canadian C-Spine Rules |
|  | Cameron (1999)  **Country:**  Canada  **Study design:**  Before and after study |  | 63 Ontario hospitals including 5 smaller community hospitals, 5 larger community hospitals, 4 larger community hospitals and 1 teaching hospital | 407 clinicians   - Emergency nurses - Family physicians - Other healthcare professionals (e.g. physiotherapists, x-ray technicians and managers) | Adults (aged 18 years or older) presenting with an ankle injury caused by acute blunt trauma (including twisting, falling and direct blows)  Sample size unclear | Not reported | Ottawa Ankle Rules |
|  | Cevik (2019)  **Country:**  Turkey  **Study design:**  Cross-sectional observational study |  | Survey of family physicians providing primary care services (Teaching hospital, Family and community health center, State hospital, Private hospital/clinic) | 456 clinicians   - Family medicine (FM) resident - General practitioner - Family medicine specialist - Contracted family medicine specialty students - Professor of FM - Assistant Professor of FM - Associate Professor of FM | N/A | **Clinicians**   - Females: n=307 (67.3%) - Median age: 30 years - Mean time since graduation: 7.28 ± 6.87 years | Ottawa Ankle Rules |
|  | Chilvers (2018)  **Country:**  England  **Study design:**  Cross-sectional observational study |  | 18 major trauma centres across England | 18 Lead trauma centres | N/A | Not reported | NEXUS & Canadian C-Spine Rules |
|  | Clement (2011)  **Country:**  Canada  **Study design:**  Cross-sectional observational study |  | Survey of ED nurses in six hospitals | 137 Emergency nurses | N/A | **Clinicians**   - Females: n=109 (79.6%) - Worked in tertiary care practice: n=92 (67.2%) - Full-time employed: n=80 (58.4%) | Canadian C-Spine Rules |
|  | Clement (2016)  **Country:**  Canada  **Study design:**  Cross-sectional observational study |  | Survey of ED staff of nine large teaching hospitals in  Ontario, in six regional trauma centres | 456 Clinicians   - ED triage nurses, - ED physicians and - Administrators | N/A | **Clinicians**   - ED triage nurses: n=281 - ED Physicians: n=151 - Administrators: 24   **ED triage nurses (n=281)**   - Females: n=251 (80.9%)   **ED triage nurses employment status**   - Full-time: 51.4% - Part- time: 17.5% - Unknown: 31.1%   **ED triage nurses highest education level**   - Masters (Nursing or other): 4.3% - Baccalaureate: 54.4% - Diploma: 36.3% - Specialty education: 10.3% | Canadian C-Spine Rules |
|  | Dowling (2011)  **Country:**  Canada  **Study design:**  Cross-sectional observational study |  | Survey of pediatric emergency physicians across Canada | 144 physician members of Pediatric Emergency Research Canada (PERC) | N/A | **Clinicians (n=144)**   - Years in practice (SD): 9.24 (6.8) - Full time practice: n=109 (75.7%) - Primary practice paediatrics: n= 104 (72.2%) | Ottawa Ankle Rules |
|  | Eagles (2008)  **Country:**  Australasia, UK, Canada, USA  **Study design:**  Cross-sectional observational study |  | Survey of members from emergency physician associations across four countries. | 1297 members of four emergency physician associations. | N/A | **Clinicians**  **Mean age (SD)**   - Australasia: 42 (6.3) years - UK: 42 (8.6) years - Canada: 42 (8.1) years - USA: 46 (8.0) years   **Females: n=278 (24.2%)**   - Australasia: n=113 (27%) - UK: n=45 (29%) - Canada: n=75 (22%) - USA: n=45 (19%)   **≥1 Emergency medicine credentials: n=1076 (83%)**   - Australasia: n=417 (100%) - UK: n=148 (96%) - Canada: n=281 (83%) - USA: n=225 (94%)   **Median (IQR) years of practice**   - Australasia: 10 (8-15) years - UK: 9 (6-15) years - Canada: 10 (5-16) years - USA: 15 (9-21) years   **Median (IQR) number of cervical spine injuries seen per month**   - Australasia: 10 (6-20) - UK: 10 (6-20) - Canada: 10 (6-18) - USA: 20 (10-30)   **Median (IQR) number of head injuries seen per month**   - Australasia: 15 (10-20) - UK: 20 (10-30) - Canada: 10 (5-15) - USA: 15 (8-28)   **Full-time employment in ED: n=904 (70%)**   - Australasia: n=346 (83%) - UK: n=90 (58%) - Canada: n=258 (76%) - USA: n=210 (88%)   **Teaching hospital: n=886 (68%)**   - Australasia: n=380 (91%) - UK: n=140 (90%) - Canada: n=251 (74%) - USA: n=115 (48%)   **Non-teaching hospital: n=266 (21%)**   - Australasia: n=38 (9%) - UK: n=16 (10%) - Canada: n=88 (26%) - USA: n=124 (52%)   **Availability of CT 24 hours a day: n=1048 (81%)**   - Australasia: n=388 (93%) - UK: n=152 (98%) - Canada: n=271 (80%) - USA: n=237 (99%) | Canadian C-Spine Rules & Canadian CT Head Rules |
|  | Gomes (2020)  Australia  **Study design:**  Retrospective review of charts |  | A tertiary hospital in South Australia | Clinicians:   - Consultants - Registrars/medical officers/interns - physiotherapists - nurse practitioners.   Sample size unclear | 262 Adults (aged 18 years or older) presenting with an acute ankle injuries to the emergency department | **Patients (n=262)**   - Mean age (SD): 38 (18.3) years - Females: n=130 (49.6%)   **Duration of injury: n=262 (100%)**   - None specified: n=200 (76.3%) - <24h: n=24 (9.2%) - 1-7 days: n=34 (13.0%) - 7-14 days: n=4 (1.5%)   **Mechanism of injury: n=262 (100%)**   - Falls: n=40 (15.3%) - Motor vehicle accident: n=3 (1.1%) - Sporting injury: n=23 (8.8%%) - Inversion/eversion injury:n=85 (32.4%) - Other (i.e., rolling, twisting): n=111 (42.4%)   **Clinicians**  Not reported | Ottawa Ankle Rules |
|  | Graham (1998)  **Country:**  Canada  **Study design:**  Cross-sectional observational study |  | Survey of members of the Canadian Association of Emergency Physicians | 232 members of the Canadian Association of Emergency Physicians | N/A | **Clinicians**   - Mean age (SD, range): 37.5 (7.1, 25-60) years - Females: n= 46 (19.8%)   **Experience and work setting**   - Emergency medicine credentials: n=155 (66.8%) - Full-time employed: n=163 (70.2%) - Mean (SD) number of hours devoted to direct patient care per week: 29.6 (11.4) - Community hospital setting: n=107 (46.1%) - Teaching hospital setting: n=125 (53.9%) | Ottawa Ankle Rules & Ottawa Knee Rule |
|  | Graham (2001)  **Country:**  Canada, USA, UK, France, Spain  **Study design:**  Cross-sectional observational study |  | Survey across five countries | 1769 emergency physicians | N/A | **Clinicians**  **Mean age (SD)**   - Canada: 38 (7) years - USA: 41 (8) years - UK: 42 (9) years - France: 40 (8) years - Spain: 39 (5) years   **Females: n=421 (24%)**   - Canada: n=84 (22%) - USA: n=38 (16%) - UK: n=70 (23%) - France: n=158 (28%) - Spain: n=71 (26%)   **Mean (SD) years since graduation**   - Canada: 11 (7) - USA: 14 (9) - UK: 18 (9) - France: 11 (8) - Spain: 13 (5)   **Full-time employment in ED: n=1412 (80%)**   - Canada: n=250 (65%) - USA: n=194 (81%) - UK: n=276 (90%) - France: n=470 (83%) - Spain: n=222 (81%)   **≥1 Emergency medicine credentials: n=1190 (72%)**   - Canada: n=280 (73%) - USA: n=222 (93%) - UK: n=288 (94%) - France: n=487 (86%) - Spain: Not applicable   **Mean (SD) year practising emergency medicine**   - Canada: 9 (7) - USA: 10 (8) - UK: 10 (7) - France: 9 (6) - Spain: Not asked   **Mean (SD) number of hours worked in emergency medicine per week**   - Canada: 29 (11) - USA: 39 (13) - UK: 29 (14) - France: 34 (19) - Spain: 43 (14)   **Teaching hospital (university or community): n=751 (42%)**   - Canada: n=238 (62%) - USA: n=129 (54%) - UK: n=110 (36%) - France: n=153 (27%) - Spain: n=121 (44%)   **Community, general, district general, hospitals: n=847 (48%)**   - Canada: n=142 (37%) - USA: n=108 (45%) - UK: n=196 (64%) - France: n=357 (63%) - Spain: n=44 (16%)   **Ambulance service: n=82 (30%)**   - Canada: - - USA: - - UK: - - France: - - Spain: n=82 (30%)   **Other setting: n=38 (2%)**   - Canada: n=4 (1%) - USA: n=2 (1%) - UK: - - France: n=57 (10%) - Spain: n=27 (10%) | Ottawa Ankle Rules & Ottawa Knee Rule |
|  | Gravel (2010)  **Country:**  Canada  **Study design:**  Randomised controlled trial |  | A paediatric ED located in urban, tertiary care, Level 1 trauma centre, Montreal, Quebec | 95 clinicians   - Medical students - Specialty residents (family medicine, emergency medicine, paediatric, other) | N/A | **Clinicians**  Females: n=74 (78%)  Medical students: n=49 (51%)  Specialty residents: n=46 (48%)   - Family medicine: n=33 (72%) - Emergency medicine: n=3 (6%) - Paediatric: n=10 (22%) | Ottawa Ankle Rules |
|  | Griffith (2011)  **Country:**  USA  **Study design:**  Retrospective review of charts |  | Level 1 adult trauma centre, Detroit, Michigan | - Emergency physicians - Radiology residents   Sample size unclear | 1589 adults who underwent CT screening of the cervical spine as part of an evaluation for blunt trauma | **Patients**   - Mean age (range): 43.4 (18-100) years - Females: n=631 (40.7%)   **Mechanism of injury: n=1589 (100%)**   - Motor vehicle crash: n=599 (37.7%) - Assault: n=477 (30%) - Falls: n=381 (24%) - Pedestrian versus motor vehicle: n=70 (4.4%) - Variety of miscellaneous injuries: n=62 (3.9%)   **Clinicians**  Not reported | NEXUS |
|  | Griffith (2013)  **Country:**  USA  **Study design:**  Prospective Study |  | Level 1 adult trauma centre, Detroit, Michigan | - Emergency physicians - Junior doctors - Physician assistants   Sample size unclear | 507 adults who underwent CT screening of the cervical spine as part of an evaluation for blunt trauma | **Patients**   - Mean age (range): 44 (18-100) years - Females: n=198 (39.1%)   **Mechanism of injury: n=1589 (100%)**   - Motor vehicle crash: n=203 (40%) - Falls: n=150 (29.6%) - Assault: n=99 (19.5%) - Pedestrian versus motor vehicle: n=23 (4.5%) - Other/not recorded: n=32 (6.4%)   **Survey completed for patients by clinician type**   - Resident physicians: n=301 (59.4%) - Senior staff physicians: n=115 (22.7%) - Physician assistants: n=45 (8.9%) - No documentation of completing personnel: n=46 (9.1%)   **Clinicians**  Not reported | NEXUS & Canadian C-Spine Rules |
|  | Hopkins (2010)  **Country:**  UK  **Study design:**  Retrospective observational study |  | One Emergency Department in Leicester | - Senior House Officers  - Emergency Nurse Practitioners  Sample size unclear | 60 adults (aged 18 years or older) presenting with an ankle injury | **Patients**   - Mean age (range): 38.82 (18-87) years - Females: n=29 (48%) | Ottawa Ankle Rules |
|  | Huang (2013)  **Country:**  China  **Study design:**  Cross-sectional observational study |  | Several hospitals in China | 247 Emergency physicians | N/A | **Clinicians**   - Females: n=72 (29.1%) - Head CT available anytime: n=232 (93.9%) - Teaching hospital: n=139 (56.3%) - Received specific training regarding radiation dose of CT: n=88 (35.6%)   **Years of practice: n=247 (100%)**   - >15 years: n=9 (3.6%) - 11-15 years: n=62 (25.1%) - 6-10 years: n=115 (46.6%) - ≤5 years: n=61 (24.7%)   **Current professional rank: n=247 (100%)**   - Attending physician: n=65 (26.3%) - Fellow: n=122 (49.4%) - Resident: n=60 (24.3%)   **Primary training: n=247 (100%)**   - Medicine: n=109 (44.1%) - Surgery: n=98 (39.7%) - Emergency medicine: n=40 (16.2%) | Canadian CT Head Rules |
|  | Knox (2015)  **Country:**  Australia  **Study design:**  Cross-sectional observational study |  | Survey of 10 universities across five states and territories in Australia. | 211 clinical educators | N/A | **Clinical educators**   - Females: n=146 (69%)   **Age**   - ≤25 years: n=6 (3%) - 26-30 years: n=54 (26%) - 31-40 years: n=72 (34%) - 41+ years: n=78 (37%) - Missing data: n=1 (0%)   **Experience and work setting**   - No post-professional qualifications: n=167 (79%) - Tertiary teaching hospital: n=89 (42%) - Secondary referral hospital: n=52 (25%) - Primary health facility: n=28 (13%) - Community centre: n=26 (12%) - Private practice: n=21 (10%) - Aged care facilities: n=10 (5%) - Not-for-profit organisation: n=1(0%) - Supervised students from other universities in addition to the University of Newcastle: n=180 (85%) [range 1-10 other universities, mean (SD): 2.6 (1.32)] | Ottawa Ankle Rules & Ottawa Knee Rule |
|  | Lau (2013)  **Country:**  Australia  **Study design:**  Prospective observational study |  | One mixed adult and paediatric, urban district ED in Melbourne, Victoria | - Emergency nurse practitioners  - Emergency physicians  Sample size unclear | 174 adults presenting with an ankle and/or midfoot injury <10 days old | **Patients**   - Mean age (range): 26.4 (2-71) - Adult: n=106 (60.9%) - Children: n=68 (39.1%) - Females: n=80 (46%)   **Mechanism of injury: n=174 (100%)**   - Fall: n=49 (28.2%) - Sport: n=42 (24.1%) - Twisting: n=69 (39.7%) - Trauma: n=14 (8.0%)   **Clinicians**  Not reported | Ottawa Ankle Rules |
|  | Ngatchou (2018)  **Country:**  Belgium  **Study design:**  Retrospective observational study |  | One emergency department in Massachusetts | Emergency physicians  Sample size unclear | 281 patients aged 16 years or older presenting to ED following blunt trauma and who underwent cervical spine radiography | **Patients**   - Mean age: 38.6 years - Females: n=131 (46.6%) - High mechanism of trauma: n=199 (70.8%) - Local pain: n=112 (40.6%)   **Clinicians**   - Not reported | NEXUS & Canadian C-Spine Rules |
|  | O'Sullivan (2006)  **Country:**  Ireland  **Study design:**  Before and after study |  | One Emergency Department of Mercy University Hospital, Cork | Emergency physicians  Sample size unclear | 43 adults presenting to the emergency department with an acute knee injury | **Patients**  **Pre-intervention** **period, n=43**  **Clinicians**  Not reported | Ottawa Knee Rule |
|  | Ozan (2018)  **Country:**  Turkey  **Study design:**  Cross-sectional observational study |  | Survey of clinicians (department chiefs, specialists, and residents) working in university, government, and private hospitals in charge of evaluating MHI patients across Turkey | 607 clinicians  - Emergency physician: n=201  - Neurosurgeons: n=179  - Radiologists: n=227 | N/A | **Clinicians**  **Experience and work setting**   - Worked in university hospitals: n=200 (32.9%) - Worked in education and research hospitals: n=159 (26.2%) - Worked in state hospitals: n=140 (23.1%) - Worked in private practice: n=108 (17.8%) | Canadian CT Head Rules |
|  | Paxton (2012)  **Country:**  Australia  **Study design:**  Retrospective review of charts |  | Regional centre in Northern Queensland |  | 406 adult patients who underwent cervical spine radiography for investigation of acute cervical spine injury secondary to trauma | **Patients**   - Mean age (range): 38 (18-98) years - Females: n=162 (40%)   **Dangerous mechanism of injury: n=196 (100%)**   - Fall from elevation: n=47 (24%) - Motor vehicle collisions: n=113 (57.6%) - Others: n=36 (18.4%)   **Clinicians**  Not reported | Canadian C-Spine Rules |
|  | Rostas (2015)  **Country:**  USA  **Study design:**  Before and after study |  | Combined adult and paediatric Level I trauma center, Richmond, Virginia | - Adult trauma surgeons  - Emergency physicians  Sample size unclear | 128 children <15 years old. First-tier or second-tier trauma team alerts | **Patients**  **Pre-intervention period, n=128**   - Median age (IQR): 6.0 (7.0) - Median Injury Severity Score (IQR), 0-75 scale: 4.0 (9.0)   **Clinicians**   - Not reported | NEXUS |
|  | Silveira (2016)  **Country:**  USA  **Study design:**  Before and after study |  | One urban academic hospital in Boston | Emergency physicians  Sample size unclear | 205 ED visits for 457 adults (aged 18 years or older) presenting with an acute blunt ankle injury (within 10 days) | **Patients**  **Pre-intervention period, n=205**   - Mean age (SD): 38.5 (15.9) - Females: n=142 (69.3%) - Malleolar zone pain: n=152 (74.2%) - Midfoot zone pain: 88 (42.9%)   **Mechanism of injury: n=205 (100%)**   - Twist: n=174 (84.9%) - Fall from height: n=17 (8.3%) - Direct blow: n=11 (5.4%) - Motor vehicle crash: n=2 (1.0%) - Other: n=1 (0.5%)   **Clinicians**  Not reported | Ottawa Ankle Rules |
|  | Slaar (2016)  **Country:**  Netherlands  **Study design:**  Retrospective observational study |  | One combined adult and pediatric level-one university trauma centre with a paediatric surgery department in Rotterdam | Emergency physicians  Sample size unclear | 573 children presenting to ED following blunt trauma | **Patients**   - Median age (IQR): 11 (5.25-15) years - Females: n= 237 (41.3%) - Fall from height: n=332 (58%)   **Clinicians**  Not reported | NEXUS |
|  | Tajmir (2017)  **Country:**  USA  **Study design:**  Randomised controlled trial |  | One urgent care centre affiliated with quaternary care at an academic hospital in Boston | 66 clinicians  - Medical doctors  - Physician assistants | 374 patient visits with an ankle or foot injury | **Patients**   - Seen by a medical doctor: n=265 (71%) - Seen by physician assistants: n=109 (29%)   **Clinicians**  **Initial randomisation, n=34 (9%)**   - Medical doctor: n=24 (71%) - Physician assistants: n=10 (29%)   **Clinicians that saw patients, n=16 (4%)**   - Medical doctor: n=12 (75%) - Physician assistants: n= 4 (25%)   **Mean years of experience, n=16**   - Medical doctor: 18.5 - Physician assistants: 9.3   **Females: n=7 (44%)**   - Medical doctor: n=3 (25%) - Physician assistants: n=4 (100%) | Ottawa Ankle Rules |
|  | Tan (2018)  **Country:**  Singapore  **Study design:**  Retrospective observational study |  | One Emergency department at National University Hospital in Singapore | Emergency physicians  Sample size unclear | 349 patients aged 16 years or older presenting to the ED following blunt trauma | **Patients**   - Mean age (range) 48 (30-68) years - Females: n=131 (37.5%)   **Mechanism of injury: n=349 (100%)**   - Fall: n=207 (59.3%) - Motor vehicle crash: n=59 (16.9%) - Assault: n=42 (12%) - External environment: n=22 (6.3%) - Sports: n=18 (5.2%) - Other: n=1 (0.3%)   **Clinicians**   - Not reported | Canadian CT Head Rules |
|  | Vedin (2017)  **Country:**  Sweden  **Study design:**  Prospective observational study |  | One Emergency Department at Helsingborg General Hospital | 69 speciality physicians who manage patients with head trauma   - ENT - Surgery - Emergency medicine - Internal medicine - Other | 161 adults presenting to the ED following blunt trauma | **Patients**   - Mean age (SD): 51.1 (21.64) years - Females: n= 61 (38%) | Canadian CT Head Rules |
|  | Wynn-Thomas (2002)  **Country:**  New Zealand  **Study design:**  Cross-sectional observational study |  | Survey across Wellington and Christchurch after-hours medical centres. | 410 general practitioners | 200 adults (aged 18 years or older) presenting with an injury to the ankle and/or foot region | **Patients**   - Females: n= 106 (53%)   **Clinicians**   - Not reported | Ottawa Ankle Rules |
|  | Zakhari (2016)  **Country:**  USA  **Study design:**  Before and after study |  | One acute care academic facility in New York | 100 clinicians   - Nurse practitioners: n=25 - Physician assistants: n=7 - Attending physicians: n=11 - Postgraduate years 1: n=1 - Postgraduate years 2: n=5 - Postgraduate years 3: n=2 - Registered nurses: n=49 | N/A | **Clinicians**   - Not reported | Canadian CT Head Rules |

A&E: Accident & Emergency; CT: Computed Tomography; ED: Emergency Department; ENT: Ear-Nose-Throat; IQR: Inter Quartile Range; NEXUS: National Emergency X-Radiography Utilization Study; SD: standard deviation; UK: United Kingdom; USA: United States of America.

* The study only provided demographics for the total sample (n=60) before and after an implementation intervention

**Supplementary Table 2: Methodological quality ratings of included studies using a modified ‘Downs and Black’ checklist**

|  | Article | 1. Is the hypothesis/  aim/objective of the study clearly described? (0,1) | 2. Are the main outcomes to be measured clearly described in the Introduction or Methods section? (0,1) | 3. Are the characteristics of the patients included in the study clearly described? (0,1) | 4. Are the main findings of the study clearly described? (0,1) | 5. Were the subjects asked to participate in the study representative of the entire population from which they were recruited? (e.g. random sampling) (0,0,1) | 6. Were those subjects who were prepared to participate representative of the entire population from which they were recruited? (0,0,1) | 7. Were the statistical tests used to assess the main outcomes appropriate? (assume appropriate if unable to determine) (0,0,1) | 8. Were the main outcome measures used accurate (valid and reliable)? Score yes if there was some attempt to validate their survey or data collection tool (0,0,1) | Total Score |
| --- | --- | --- | --- | --- | --- | --- | --- | --- | --- | --- |
| 1 | Allerston (2000) | 1 | 1 | 1 | 1 | 1 | 1 | 1 | 1 | 8 |
| 2 | Ashurst (2014) | 1 | 1 | 1 | 1 | 1 | 1 | 1 | 1 | 8 |
| 3 | Belot (2017) | 1 | 1 | 1 | 1 | 1 | 0 | 1 | 1 | 7 |
| 4 | Benayoun (2016) | 1 | 1 | 1 | 1 | 1 | 1 | 1 | 1 | 8 |
| 5 | Bessen (2009) | 1 | 1 | 1 | 1 | 0 | 1 | 1 | 1 | 7 |
| 6 | Beutel (2012) | 1 | 1 | 1 | 1 | 1 | 0 | 1 | 0 | 6 |
| 7 | Borg (2008) | 1 | 1 | 1 | 1 | 1 | 1 | 1 | 1 | 8 |
| 8 | Brehaut (2005) | 1 | 1 | 1 | 1 | 1 | 0 | 1 | 1 | 7 |
| 9 | Brehaut (2006) | 1 | 1 | 1 | 1 | 1 | 1 | 1 | 1 | 8 |
| 10 | Burns (2011) | 1 | 1 | 1 | 1 | 1 | 0 | 1 | 1 | 7 |
| 11 | Cameron (1999) | 1 | 1 | 1 | 1 | 1 | 0 | 1 | 1 | 7 |
| 12 | Cevik (2019) | 1 | 1 | 1 | 1 | 0 | 0 | 1 | 1 | 6 |
| 13 | Chilvers (2018) | 1 | 1 | 0 | 1 | 1 | 1 | 1 | 0 | 6 |
| 14 | Clement (2011) | 1 | 1 | 1 | 1 | 1 | 1 | 1 | 1 | 8 |
| 15 | Clement (2016) | 1 | 1 | 1 | 1 | 1 | 1 | 1 | 1 | 8 |
| 16 | Dowling (2011) | 1 | 1 | 1 | 1 | 1 | 1 | 1 | 1 | 8 |
| 17 | Eagles (2008) | 1 | 1 | 1 | 1 | 1 | 0 | 1 | 0 | 6 |
| 18 | Gomes (2020) | 1 | 1 | 1 | 1 | 1 | 1 | 1 | 0 | 7 |
| 19 | Graham (1998) | 1 | 1 | 1 | 1 | 1 | 1 | 1 | 0 | 7 |
| 20 | Graham (2001) | 1 | 1 | 1 | 1 | 1 | 0 | 1 | 1 | 7 |
| 21 | Gravel (2010) | 1 | 1 | 1 | 1 | 0 | 1 | 1 | 1 | 7 |
| 22 | Griffith (2011) | 1 | 1 | 1 | 1 | 1 | 1 | 1 | 1 | 8 |
| 23 | Griffith (2013) | 1 | 1 | 1 | 1 | 1 | 1 | 1 | 1 | 8 |
| 24 | Hopkins (2010) | 1 | 1 | 1 | 1 | 0 | 1 | 1 | 1 | 7 |
| 25 | Huang (2013) | 1 | 1 | 0 | 1 | 1 | 0 | 1 | 0 | 5 |
| 26 | Knox (2015) | 1 | 1 | 1 | 1 | 0 | 1 | 1 | 1 | 7 |
| 27 | Lau (2013) | 0 | 1 | 1 | 1 | 1 | 1 | 1 | 1 | 7 |
| 28 | Ngatchou (2018) | 1 | 1 | 1 | 1 | 1 | 0 | 1 | 1 | 7 |
| 29 | O'Sullivan (2006) | 1 | 1 | 1 | 0 | 0 | 0 | 1 | 0 | 4 |
| 30 | Özan (2018) | 1 | 1 | 1 | 1 | 0 | 0 | 1 | 0 | 5 |
| 31 | Paxton (2012) | 1 | 1 | 1 | 1 | 1 | 1 | 1 | 1 | 8 |
| 32 | Rostas (2015) | 1 | 1 | 1 | 1 | 0 | 0 | 1 | 1 | 6 |
| 33 | Silveira (2016) | 1 | 1 | 1 | 1 | 1 | 0 | 1 | 1 | 7 |
| 34 | Slaar (2016) | 1 | 1 | 1 | 1 | 1 | 1 | 1 | 1 | 8 |
| 35 | Tajmir (2017) | 1 | 1 | 1 | 1 | 1 | 0 | 1 | 1 | 7 |
| 36 | Tan (2018) | 1 | 1 | 1 | 1 | 1 | 1 | 1 | 1 | 8 |
| 37 | Vedin (2017) | 1 | 1 | 1 | 1 | 1 | 1 | 1 | 1 | 8 |
| 38 | Wynn-Thomas (2002) | 1 | 1 | 1 | 1 | 0 | 1 | 1 | 1 | 7 |
| 39 | Zakhari (2016) | 1 | 1 | 1 | 1 | 0 | 0 | 1 | 1 | 6 |

**Supplementary Table 3. Summary of results from included studies**

| **Author (year)** | **Self-reported use** | **Documentation of use of rules or documentation of clinical features consistent with rules** | **Awareness or attitudes to use of rules** | **Other outcomes** |
| --- | --- | --- | --- | --- |
| **Cervical Spine Rule and NEXUS** | | | | |
| Belot (2017) | Not reported | **Documentation of clinical features consistent with rules**  Physiotherapists: n= 362/457 (79%) | **Physiotherapists’ awareness of the C-spine rule**  Heard about the rule: n= 193/457 (42%)  **Attitude towards C-spine rule after being exposed to it n=457**   - Consider using the rule in their practice: n= 445/457 (97%) - Did not see any barriers to using the rule in the practice: n= 407/457 (89%)   **Barriers to implementing in practice: n= 50/457 (11%)**   - it was too rigid, - it addressed content outside the physiotherapy scope of practice, - they were afraid of missing serious injuries, - they believed that clinical judgment was as good or better, and - they might forget the details of - the rule.   **Facilitators (n= 457)**   - Having one or more laminated flowcharts in their office: n= 408/457 (89%) - having access to the rule on the Physiotherapy Association of British Columbia website: n= 255/457 (56%) - viewing live or video depictions of the rule in use in patient simulations: n= 188/457 (41%) - receiving occasional email reminders about the rule: n= 122/457 (27%) - having the ability to contact a knowledgeable physiotherapist for advice: n= 87/457 (19%) - having access to the rule through a cell phone app, learning the rule in pre-licensure training, including in postgraduate clinical orthopaedic courses, and being able to refer directly for imaging rather than through a physician: n= 16/457 (4%) | Not reported |
| Benayoun (2016) | Not reported | **NEXUS:**   - Appropriate imaging: n= 522/760 patients (69%) - Inappropriate imaging: n= 167/760 patients (22%) - Indeterminate: n= 71/760 patients (9%)   **CCR:**   - Appropriate imaging: n= 376/760 patients (50%) - Inappropriate imaging: n= 157/760 patients (21%) - Indeterminate: n= 227/760 patients (30%) | Not reported | **Referred for CT w/o indication: n= 167**  Admitted: n= 66 (40%)  Head CT performed: n= 154 (92%)  No acute intracranial abnormality” n= 43 of 66 admitted (65%)  **Detection of fractures: n= 7**  **Patients found to have fractures who were not given an X-ray: n= 0** |
| Brehaut (2006) | **Self-reported use of CCR by emergency physicians n= 165/219 (75%)**   - Using always or most of the time: n= 150/262 (57%) | Not reported | **Awareness of CCR:**   - Aware of the rule: n= 219/262 (84%) - Aware and already using the rule in their practice: n= 165/219 (75%) - Never seen the rule: n=43/262 (17%) - Seen but do not use the rule: n=54/262 (21%)   **Attitudes of CCR:**   - Not currently using the rule but considering using the rule in the future: n= 72/97 (74%) - Not consider using the rule in the future: n=19/97 (20%)   **Ratings of C-Spine versus the OAR**  **C-Spine Rule**   - Easy to learn 74% - Easy to remember 60% - Easy to use 76% - Efficient use of time 81% - Useful in my practice 88% - Too unsafe 3% - Too much trouble to apply 6% - Increase the chance of lawsuits 2%   **CCR attitudes**   - Easy to learn 74% - Easy to remember 60% - Easy to use 76% - Efficient use of time 81% - Useful in my practice 88% - Too unsafe 3% - Too much trouble to apply 6% - Increase the chance of lawsuits 2%   **Barriers to not using the CCR: n= 19**   - Rule was too complicated: n= 12/19 (63%) - Working in an only pediatric patient practice: n= 3/19 (16%) - use of a competing rule: n= 1/19 (5%) - resistance to a change in practice: n= 1/19 (5%) - concern over specific aspects of the rule: n= 1/19 (5%) - concerns over the validation of the rule: n= 1/19 (5%) |  |
| Burns and Yanchar (2011) | **Self-reported use of guidelines (NEXUS, CCSR, or locally developed) by pediatric emergency physicians and trauma team Leaders n= 155**   - Reported use ‘almost always’ or ‘most of the time’ for patients <16yrs: n= 94/155 physicians (61%) - <50% of the time (occasionally): n= 7/155 (5%) - 0-10% of the time (almost never): n= 53/155 (34%)   **Guidelines used:**   - NEXUS: n= 20/87 physicians (23%) - CCR: n= 21/87 physicians (24%) - locally developed guidelines: n= 36/87 physicians (41%) - Multiple sources: n= 10/87 physicians (12%) | Not reported | Would not use a guideline even if it were  made available: n=11/53 (14%)  Barriers to not using the rule n=11 physicians   - Not enough research to support the use of a guideline: n= 7 (64%) - clinical protocols are not useful in general: n= 2 (18%) - preferred to use their own judgment: n= 1 (9%), - it would depend on the guideline: n= 1 (9%)   **Barriers to not using the rule n=53 physicians (almost never used a guideline to clear pediatric C-spines)**   - unaware of any guidelines produced at their centres: n= 27/53 (50%) - Not enough evidence for patients <16yrs: n= 16/53 physicians (31%) | **Type of imaging used first:**  ‘Almost always’ use plain radiographs: n= 147/155 (95%)  If already planning CT of the head only: n= 33/155 physicians (21%) would use CT of the C-spine ‘almost always’ or ‘most of the time’ |
| Chilvers (2018)^b^ | **Self-reported use of guidelines:**  Written protocol: n= 15/17 trusts (82%)   - CCR: n= 12/17 trusts (71%) - NEXUS: n= 2/17 trusts (12%) - NEXUS & pain-free neck movement: n= 1/17 trusts (6%) - No tool used: n= 2/17 trusts (12%) | Not reported | Not reported | **First type of imaging used on C-spine:**  CT: n= 12/17 trusts (72%)  Three-view plain radiographs: n= 5/13 trusts (39%)  **Choice in initial imaging:**  Mechanism of injury and clinical assessment: n= 11/17 trusts (65%)  Diagnostic accuracy of imaging modality: n= 6/17 (35%)  Age of patient/ Radiation concerns: n= 9/17 (52%) |
| Clement (2011) | Not reported | Not reported | **Facilitators:**   - Like to try new things at work: n= 132/137 nurses (96%) - Easy for me to take on new responsibilities: n= 132/137 nurses (96%) - Like being involved in research projects that can improve patient care: n= 130/137 nurses (95%) - There are nurse role models/leaders in my unit that will advocate the use of C-spine rule: n= 129/137 nurses (94%) - It is/would be useful to my practice: n= 127/137 nurses (93%) - This rule is/would be easy to learn: n= 125/137 nurses (91%) - It is/would be an efficient use of my time: n= 125/137 nurses (91%) - Our department is used to changing practices and procedures based on new evidence: n= 121/137 nurses (88%) - Respondents comfortable applying the rule: n= 122/137 nurses (89%). - It is/would be easy to use: n= 120/137 nurses (88%) - It is/would be easy to remember: n= 118/137 nurses (86%) - My manager/administrator supports nurses’ use of the C-Spine Rule: n= 116/137 nurses (85%) - Nurses’ application of the C-Spine Rule will be a big help in making the ED more efficient: n= 114/137 nurses (83%) - Our doctors trust the nurses’ ability to do the C-Spine Rule: n= 98/137 nurses (72%) - When nurses do the Rule, doctors will be able to concentrate on more serious cases: n= 94/137 nurses (69%) - There will be fewer complaints from our patients when we apply the C-Spine Rule: n= 91/137 nurses (66%)   **Barriers:**   - Heavy workload makes it hard to apply to C-Spine Rule: n= 51/137 nurses (37%) - Increase the chance of lawsuits: n= 50/137 nurses (37%) - Belief that it is not nurses’ role to apply the C-Spine Rule: n= 38/137 nurses (28%) - It takes time for me to change the way I care   for my patients: n= 34/137 nurses (25%)   - It is/would be too rigid to apply to individual patients: n= 27/137 nurses (20%) - It is/would be too unsafe: n= 26/137 nurses (19%) - It is/would be too much trouble to apply: n= 19/137 nurses (14%) - It is hard for me to learn new ways of doing things: n= 15/137 nurses (11%)   **Local facilitators and barriers**  **Local facilitators**   - Good support and reinforcement from physicians in the ED: n= 37/137 (27%) - Good support and guidance from nurse educators/managers in the ED: n= 27/137 (20%) - Good peer support and cooperation of all nursing staff: n= 27/137 (20%) - Frequent refresher sessions on the C-Spine Rule: n=24/137 (18%) - Many visual aids such as posters on the Canadian C-Spine Rule: n= 14/137 (10%) - Access to local ‘‘champion’’ nurse in the ED: n= 10/137 (7%)   **Local barriers**   - Some staff lack time at triage to do rule/ED department is too busy: n= 53/137 (39%) - Fear of making a mistake with the C-Spine rule/litigation issues: n= 19/137 (14%) - Fear of change in nursing practice/ negative attitude: n= 16/137 (12%) - Forgot to use the C-Spine Rule: n= 9/137 (7%) - Difficulty interpreting ‘‘dangerous’’ mechanism of injury in the rule: n= 6/137 (4%) |  |
| Clement (2016) | Not reported | Not reported | **Nurses**  **Barriers to using CCR (n=281):**   - Too busy/lack of time/workload n=101 (35.9%) - Not enough trauma patients to get comfortable using CCR n=59 (21.0%) - Physicians not on-board n=37 (13.2%) - No support/cannot find anyone to consult with n=21 (7.5%) - Patient refusal/compliance n=18 (6.4%) - Confusing rule/misinterpretation n=11 (3.9%) - Remembering to do it n=11 (3.9%) - Physical space n=10 (3.6%) - Lack of nurse participation n=9 (3.2%) - Cannot find the CCR form n=7 (2.5%) - Worried complications could arise n=7 (2.5%) - Paramedics questioning the nurses/resisting RN assessment n=4 (1.4%) - Delay in medical directive n=4 (1.4%) - Not for triage nurses/should be Charge RN or RN in room n=4 (1.4%) - Takes longer to triage n=4 (1.4%) - EMS using CCR in Ottawa n=4 (1.4%) - Not for nurses . . . MD should remove the collar n=3 (1.1%) - Not being able to remove the backboard n=3 (1.1%) - Requires refreshers n=3 (1.1%) - Lack of education n=3 (1.1%)   **Facilitators to using CCR (n=281):**   - Support from manager/educator n=86 (48.6%) - Teamwork between nurses/ physicians/ management n=83 (46.9%) - Excellent nurse champions n=60 (33.9%) - Reminders/emails/signs n=44 (24.8%) - Certified and senior staff encourage uncertified staff n=38 (21.5%) - Physician support n=38 (21.5%) - Education/refreshers n=35 (19.8%) - Easy to follow/easy to learn n=17 (9.6%) - Easy access to implementation sheets n=17 (9.6%) - Comfort for patients n=6 (3.4%) - Easy access to resources n=5 (2.8%)   **Physicians**  **Barriers to using CCR (n=151):**   - Busy department n=40 (26.5%) - Nurse reluctance/participation/comfort n=32 (21.2%) - Lack of education training and support n=31 (20.5%) - Physicians not comfortable with nurses clearing the c-spine n=27 (17.9%) - Not aware of any barriers n=21 (13.9%) - Not aware of project n=14 (9.2%) - Not necessary at our hospital n=12 (7.9%) - Legal/liability concerns n=8 (5.3%) - Lack of physician education n=8 (5.3%) - Variation in interpretation; of dangerous mechanism, intoxicated patients n=7 (4.6%) - Physicians using Nexus and CCR combined (Changing practice) n=6 (4.0%) - Not enough trauma patients n=6 (4.0%) - EMS roadblocks n=5 (3.3%)   **Facilitators to using CCR (n=151):**   - Nurse leader/champion/educator n=52 (34.4%) - Physician leader n=34 (22.5%) - Management/Nursing support n=26 (17.2%) - Nurse engagement n=19 (12.6%) - Physician enthusiasm and support n=15 (9.9%) - Frequent reminders, posters, signage n=13 (8.6%) - Timely training of nurses and physicians n=10 (6.6%) - Communication between MD/RN feedback and support n=9 (6.0%)   **Administration**  **Barriers to using CCR (n=24):**   - Lack of physician support n=9 (37.5%) - Non-compliance of nursing staff n=9 (37.5%) - Nurses perceive triage as too busy n=8 (33.3%) - Not enough trauma patients to gain competence n=7 (29.2%) - Time to train all staff n=7 (29.2%) - Competing with physician learners n=7 (29.2%) - Medical directive approval n=6 (25.0%) - Nurse champion – requires dedicated time/support n=5 (20.8%)   **Facilitators to using CCR (n=24):**   - Nurse champion and educators n=9 (37.5%) - Nurse engagement n=8 (33.3%) - Educational support n=8 (33.3%) - Management support n=6 (25.0%) - Physician support n=3 (12.5%) | Not reported |
| Eagles (2008)^b^ | **Self-reported use by** **Emergency physicians:**  **CCR use:**   - Australasia: n= 196/417 (47%) - Canada: n= 247/339 (73%) - UK: n= 190/302 (63%) - US: n= 72/239 (30%)   **Use other guidelines by Emergency physicians n=1297**  NEXUS guidelines: n= 93/1297 (7%)  Australasia: n= 66/417 (16%) | Not reported | **Awareness:**  CCR awareness   - Australasia: n= 392/417 (94%) - Canada: 329/339 (97%) - UK: n= 269/302 (89%) - US: 155/239 (65%)   **Attitudes:**  Among those emergency physicians not currently using rules, would consider using them in the future   - CCR: n= 430/705 (61%) | Not reported |
| Graham (1998) |  |  | **Attitudes towards use of cervical spine radiography in alert, stable trauma patients.**   - Do not agree all such patients should have cervical-spine radiography: n= 178/227 (79%) - Currently refer such patients for C-spine radiography (sometimes): n= 114/227 (50%) - Consider using C-spine rule: n= 200/227 (67%) - Only accept a rule 100% sensitive (identifies unstable fractures or dislocations): n= 150/222 physicians (67%) |  |
| Griffith (2011) | Not reported | **Documentation of clinical features consistent with rules**   - NEXUS low-risk criteria: n=1217/1589 (77%) - Liberalized NEXUS criteria (NEXUS criteria plus one item on active cervical spine rotation): n=1273/1589 (80%) - No NEXUS low-risk criteria documented n=372/1589 (23%) - No liberalized NEXUS low-risk criteria documented (NEXUS criteria plus one item on active cervical spine rotation): n= 316/1589 (20%) | Not reported | **Total cervical spine CT examination performed n=1589**   - Positive for an acute cervical spine injury: n=41/1589 (3%) - Negative for an acute cervical spine injury: n= 1524/1589 (96%) - Indeterminate findings for cervical spine injury but negative follow-up findings: n= 24/1589 (2%)   **Examinations with no acute**  **injury: n= 1524**   - No documentation of any of the five NEXUS criteria: n= 364/1524 (24%) - Documented no NEXUS criteria in addition to no record of limited or painful range of motion or posterolateral or paraspinal tenderness: n= 308/1524 (20%) |
| Griffith (2013) | Not reported | **Documentation of clinical features consistent with rules**   - NEXUS criteria: n= 426/507 patients - CCR criteria: n=297/416 patients with normal level of alertness   **Guidelines not followed**   - Staff physicians: n= 11/115 (10%) - Residents: n= 51/301(17%) - Physician assistants: n= 9/45 (20%) | Not reported | **Total cervical spine CT examination performed: n=507**   - Negative findings for an acute cervical spine injury: n= 497/507 (98%) - Positive findings for an acute cervical spine injury: n=5/507 (1%) - Indeterminate findings for cervical spine injury but negative follow-up findings: n= 5/507 (1%)   **Imaging appropriate by NEXUS criteria: n=426**   - Negative findings for an acute cervical spine injury: n= 417/426 (98%) - Positive findings for an acute cervical spine injury: n=5/426 (1%) - Indeterminate findings for cervical spine injury but negative follow-up findings: n= 4/426 (1%)   **Imaging inappropriate by NEXUS criteria: n=81**   - Negative findings for an acute cervical spine injury: n= 80/81 (99%) - Positive findings for an acute cervical spine injury: n=0/81 (0%) - Indeterminate Findings for Cervical Spine Injury but negative follow-up findings: n= 1/81 (1%)   **Imaging appropriate by abbreviated CCR criteria: n=297**   - Negative findings for an acute cervical spine injury: n= 289/297 (98%) - Positive findings for an acute cervical spine injury: n=4/297 (1%) - Indeterminate Findings for Cervical Spine Injury but negative follow-up findings: n= 4/297 (1%)   **Imaging inappropriate by abbreviated CCR criteria: n=119**   - Negative findings for an acute cervical spine injury: n= 118/119 (99%) - Positive findings for an acute cervical spine injury: n=0/119 (0%) - Indeterminate findings for cervical spine injury but negative follow-up findings: n= 1/119 (1%)   **Imaging appropriate by CCR and/or NEXUS criteria n= 469**   - Negative findings for an acute cervical spine injury n= 459/469 (98%) - Positive findings for an acute cervical spine injury n=5/469 (1%) - Indeterminate Findings for Cervical Spine Injury but negative follow-up findings n= 5/469 (1%)   **Imaging inappropriate by both NEXUS and CCR criteria n= 38**   - Negative findings for an acute cervical spine injury n= 38/38 (100%) - Positive findings for an acute cervical spine injury n=0/38 (0%) - Indeterminate findings for cervical spine injury but negative follow-up findings n=0/38 (0%) |
| Ngatchou (2018) | Not reported | **Documentation of clinical features consistent with rules**  CCR: n= 256/281 (92%)  NEXUS: n= 272/281 (97%) | Not reported | Not reported |
| Paxton (2012) | Not reported | **Documentation of clinical features consistent with CCR**  Presence of CCR criteria: n= 251/406 (62%) | Not reported | Not reported |
| Rostas (2015) | Not reported | **Documentation of clinical features consistent with NEXUS guideline**  Children assessed before guideline implementation: n= 128  Total cervical spine CT scans obtained: n= 55/128 (43%)  NEXUS criteria present: n= 76/128 (59% )   - imaging done: n= 46/76 (61%) (appropriate imaging)   No NEXUS criteria present: n= 51/128 (40%)   - imaging done n= 9/51 (18) (inappropriate imaging) - Indeterminate finding for cervical spine injury: n=1/128 (1%) | Not reported | NA |
| Slaar (2016) | Not reported | **Documentation of clinical features consistent with NEXUS guideline**  Adherence to guidelines: n= 573/573 patients (100%) | Not reported | **Primary Imaging Technique:**   - Plain radiography: n= 544/573 patients (95%) - Additional imaging performed: n= 104/573 patients (19%) - CT-scan: n= 29/573 patients (5%)   **Imaging adherence:**  Correct adherence: n= 252/573 patients (44%)  Incomplete set of radiographs obtained: n= 79/573 patients (15%)  <8yrs: n= 37/79 patients (47%)   - Superfluous number of radiations obtained: n= 223/573 patients (41%) - Multiple attempts to obtain the odontoid view: n= 145/223 patients 65% |
| **Canadian CT Head Rule** | | | | |
| Eagles (2008) | **CCHR use:**   - Australasia: n= 133/417 (32%) - Canada: n= 193/339 (57%) - UK: n= 63/302 (21%) - US: n= 29/239 (12%)   **Other head CT rule use by Emergency physicians n=137**  NICE guidelines: n= 85/137 (62%)  UK use of NICE: n= 78/ 85 (92%) |  | **CCHR awareness**   - Australasia: n= 342/417 (82%) - Canada: n= 292/339 (86%) - UK: n= 199/302 (66%) - US: n= 74/239 (31%)   **Attitudes:**  Among those emergency physicians not currently using rules, would consider using in the future  CCHR: n= 554/879 (63%) | Being employed at a teaching hospital decreases the odds of using the CCHR (OR = 0.6; 95% CI = 0.38 to 0.95). |
| Graham (1998) |  |  | **Attitudes towards CT head in patients with minor head injury:**   - Do not agree all minor head injury patients should have CT scan: n= 194/228 (85%) - Currently refer such patients for CT scan (sometimes): n= 158/228 (69%) - Consider using CT head rule: n= 196/228 (86%) - Only accept a rule 100% sensitive (neurosurgical lesions): n= 115/222 physicians (52%) |  |
| Huang (2013) | **CCHR:**  Utilization rate: n=61/247 (25%) | Not reported | **Awareness of CCHR:**  Very familiar or somewhat familiar: n= 103/247 physicians (42%)  **Attitude towards CCHR**  Consider using rule (not currently using rule): n= 127/186 (68%)  **Barriers to using CCHR:**   - Fear of malpractice: n= 91/186 (49%) - Pressure from administration to order more examination: n= 56/186 (30%) - Lack of knowledge about radiation risk of CT: n= 50/186 (27%) | Not reported |
| Ozan (2018) | **Use of head CT rules in minor head injury**   - Always: n= 42/607 (7%) - Mostly: n= 124/607 (20%) - Sometimes: n = 136/607 (22%) - Never: n = 305/607 (50%)   Emergency physicians use of CT rules n=201   - Always: n= 19/201 (10%) - Mostly: n= 87/201 (43%) - Sometimes: n = 63/201 (31%) - Never: n = 32/201 (16%)   Neurosurgeons use of CT rules n=179   - Always: n= 23/179 (11%) - Mostly: n= 30/179 (17%) - Sometimes: n = 37/179 (21%) - Never: n = 92/179 (51%)   Radiologists use of CT rules n=227   - Always: n= 3/227 (1%) - Mostly: n= 7/227 (3%) - Sometimes: n = 36/227 (16%) - Never: n = 181/227 (80%) | Not reported | **Awareness of head CT rules in minor head injury**   - Emergency physicians: n= 121/201 (60%) - Neurosurgeons: n= 50/179 (28%) - Radiologist: n= 17/227 (8%)   **Knowledge on head CT rules in minor head injury: n= 607**   - Sufficient: n= 189/607 (31%) - Insufficient: n= 201/607 (33%) - Absent: n= 217/607 (36%)   Knowledge among emergency physician: n=201   - Sufficient: n= 121/201 (60%) - Insufficient: n= 63/201 (31%) - Absent: n= 17/201 (9%)   Knowledge among neurosurgeons: n= 179   - Sufficient: n= 50/179 (28%) - Insufficient: n= 63/179 (35%) - Absent: n= 66/179 (37%)   Knowledge among radiologists: n=227   - Sufficient: n= 17/227 (8%) - Insufficient: n= 76/227 (34%) - Absent: n= 134/227 (59%) | **Decision to obtain a CT:**  Emergency physicians: n= 485/607 clinicians (80%)  **Radiation dose of CT:**  Received formal education: n= 257/607 clinicians (42%)  Knowledge of correct radiation dose during head CT: n= 154/607 clinicians (25%) |
| Tan (2018) | Not reported | **Documentation of clinical features consistent with CCHR**  Presence of CCHR criteria: n= 249/349 (71%) | Not reported | **CT of head**   - Recommended: n= 209/349 (60%) - Recommended but not performed: n= 30/349 (9%) - Performed but not recommended: n= 70/349 (20%) - Positive CT: n= 50/349 (14%) - Clinically significant positive CT: n= 42/349 (12%) |
| Vedin (2017) |  | **Documentation of use of guidelines:**   - CCHR applied: n= 54/161patients (34%) - No guidelines applied: n= 84/161 patients (52%)   More than 1 guidelines used: n= 6/161 patients (4%) |  | **Reasons for ordering CT: n=161**   - Confirm/Exclude diagnosis: n= 161/161 (100%) - Expedite diagnosis: n= 70/161 (44%) - Lack of in-hospital beds: n= 66/161 (41%) - Consultant request: n= 67/161 (42%) - General practitioner request: n= 67/161 (42%) - Colleague request: n= 69/161 (43%) - Pressure from patients/relatives: n= 68/161 (42%) - Fear of missing diagnosis: n= 79/161 (49%) - Fear of being reported: n= 70/161 (44%) - Elusive medical history: n= 72/161 (45%) - Other: n= 10/161 (6%) |
| Zakhari (2016) | Not reported | Not reported | **Knowledge of CCHR: n= 49/100 (49%)**   - Attending physician: n= 7/11 (59%) - Nurse practitioners: n= 13/25 (51%) - Physician assistants: n= 3/7 (46%) - Postgraduate year 1: n= 1/1 (75%) - Postgraduate year 2: n= 2/5 (50%) - Postgraduate year 3: n= 1/2 (25%) - Registered nurses: n= 22/49 (44%)   **Participants attitude towards adopting CCHR**   - Agreeing to adopt the tool into practice if it was being used by colleagues who were happy with-it: n= 50/100 (50%) - Participants reported moderate likelihood of adopting the CCHR into clinical practice: n=83/100 (83%) |  |
| **Ottawa Ankle Rules/ Ottawa Ankle and Foot Rules** | | | | |
| Allerston (2002） | Not reported | **Documentation of use of OAR**   - Nurse Practitioners: n= 187/196 patients (95%) - Medical staff: n= 0/158 patients (0%) | Not reported | **Referred for X-ray**   - Nurse Practitioners: n=115/187 patients (63%) - Medical staff: n=127/158 patients (80%)   **Detection of fracture**   - Nurse Practitioners: n=34/115 patients (30%) - Medical staff: n=29/127 patients (23%)   **Patients found to have fracture who were not given an X-ray**  Nurse practitioners: 4 patients |
| Ashurst (2014) | Not reported | **Documentation of use of OFAR**  Total documentation of use of OFAR: n=7/30 (23%)   - Nurses at triage: n= 1/30 patients (3%) - Clinicians (physician assistants, residents, and attending physicians) after triage: n= 6/30 patients (20%) | Not reported | **Referred for X-ray**  Control group: n= 29/30 patients (97%)  **Detection of fracture**  Control group: n= 9/30 patients (30%) |
| Bessen (2009) | Not reported | **Documentation of clinical features consistent with OAR**   - Total: n=249/458 (54%) - Tertiary hospital: n= 123/214 patients (58%) - Community hospital: n= 126/244 patients (52%) | Not reported | **Referred for X-ray**   - Tertiary hospital: n= 206/215 (96%) - Community hospital: n= 223/244 (91%)   **Detection of factures:**   - Tertiary hospital: n= 40/206 (20%) - Community hospital: n= 34/223 (15%)   **Missed fracture rate:**   - Tertiary hospital: n= 0/9 (0%) - Community hospital: n= 0/21 (0%) |
| Borg (2008)^a^ | Not reported | **Documentation of clinical features consistent with OAR**  **Ankle:**   - Indicated: n= 50/62 patients imaged (81%) - Did not fulfill OAR criteria: n= 12/62 (19%) - Fulfilled OAR criteria for ankle x-rays: n= 2/4 (50%) not x-rayed   **Foot:**   - Indicated: n= 19/28 patients imaged (68%) - Did not fulfill OAR criteria: n= 9/28 (32%) | Not reported | **Referred for X-ray n=69/73 (95%)**  **X-ray series (i.e. standard AP and lateral views) performed: n=90**  Ankle x-rays: n= 62/90 (69%)  Foot x-rays: n= 28/90 (31%)  **Reduction in imaging:**  Use of OAR would have reduced x-rays: 22%  Ankle: (19%)  Foot: (32%) |
| Brehaut (2005) | **Self-reported use of OAR by emergency physicians**   - ‘always’ or ‘most of the time: n= 235/262 (90%) - Sometimes: n= 26 (10%) - Never: n= 1 (0.4%) | Not reported | **Awareness of OAR: n= 260/262 (99%)**  Learnt about OAR **n= 253**   - medical school or residency: n= 79/253 physicians (31%) - Other awareness: n= 174/253 physicians (69%)   **Attitudes towards OAR (strongly or moderately agree) n= 258**   - easy to learn: n= 247/258 (96%) - easy to use: n= 244/258 (95%) - useful in their practices: n= 239/258 (93%) - easy to remember: n= 229/258 (89%)   **How would you describe the role of the OAR in your decision about whether to order a radiograph? (n = 258)**   - I decide primarily on the basis of the rule: n= 109/258 (42%) - Rule plus a small number of factors: n= 109/258 (42%) - Rule as one of many factors: n= 39/258 (15%) - Generally do not consider rule: n= 3/258 (1%)   **Test of memory for OAR n=262**   - correctly remember all four components: n= 138/262 (53%) - correctly remember three components: n= 69/262 (26%) - correctly remember two components: n= 42/262 (16%) - correctly remember one component: n= 10/262 (4%)   **Facilitators:**  OAR poster in ED: n= 155/255 physicians (61%)  **Barriers:**   - Gross deformity 97% - Communication problem 88% - Distracting painful injuries 76% - Swelling of medial malleolus 54% - Ankle fracture within 12 months 41% - Swelling of lateral malleolus 33% - Cracking sounds heard 30% - Fracture risk e.g. Osteoporosis 82% - Fall from height 71% - Age ($55 years) 55% - Hostile patient 66% - Patient requests radiography 38% | Not reported |
| Cameron (1999) | Not reported | Not reported | **Awareness of the OAR:** n= 289/407 (71%) | **Patients referred for ankle radiography**  Group A hospital: n= 119/162 (73%)  Group B hospital: n= 176/241 (73%)  Group C hospital: n= 181/240 (75%)  **Patients referred for foot radiography**  Group A hospital: n= 25/162 (15%)  Group B hospital: n= 45/241 (19%)  Group C hospital: n= 46/240 (19%)  **Detection of fractures**  Group A hospital: n=25/162 (15%)  Group B hospital: n= 36/241 (15%)  Group C hospital: n= 36/240 (15%) |
| Cevik (2019) |  | Not reported | **Awareness of the OAR (n=456)**   - 89/456 (19.5%)   **Physicians who evaluated all the 5 items of the OAR as an indicated for taking an X-ray:**   - 49/456 (11%) | Not reported |
| Dowling (2011) | **Self-reported use of OAR by emergency physicians**  Always or most of the time: n= 82/144 (57%) | Not reported | **Not reported** | Not reported |
| Gomes (2020) | Not reported | **Documentation of clinical features consistent with OAR**  OAR documented= 163/262 (62%) | Not reported | Compliance with the rules   - Physiotherapists: 77.3% - Nurses: 65.4% - Consultants: 61.5% - Registrars/MOs/Interns: 54.8% |
| Graham (1998) | **Self-reported use of OAR by emergency physicians**  Always or almost always: n= 190/232 (82%) | Not reported | **Physicians’ attitude towards clinical decision rules:**  Agreed or strongly agreed   - Protect against complaints: n= 99/230 physicians (43%) - Intended to cut healthcare costs: n= 178/231 physicians (77%)   Disagreed or strongly disagreed   - Oversimplified medicine: n= 157/231 physicians (68%) - Too rigid to apply to individual patients: n= 169/231 physicians (73%) - Too time-consuming to apply in the ED: n= 207/230 physicians (90%) |  |
| Graham (2001)^b^ | **Self-reported use of OAR**  **OAR use:**  Always/ Most of the time   - Canada: n= 334/375 (89%) - USA: n= 70/227 (31%) - UK: n= 215/295 (73%) - France: n= 166/535 (31%)   Spain: n= 24/270 (9%) | Not reported | **OAR awareness among emergency physicians:**   - Canada: n= 373/375 (99%) - USA: n= 217/227 physicians (96%) - UK: n= 268/295 physicians (91%) - France: n= 371/535 physicians (69%) - Spain: n= 56/270 physicians (21%)   **Facilitators:**  Agree, strongly agree %   - Indented to cut healthcare costs: USA 85%, Canada 74%, UK 36%, France 61%, Spain 71% - Good educational tool: USA 83%, Canada 85%, UK 77%, France 77%, Spain 62% - Intended to improve quality of care: USA 79%, Canada 90%, UK 88%, France 84%, Spain 81% - A convenient source of advice: USA 79%, Canada 86%, UK 84%, France 68%, Spain 88% - Likely to decrease health care costs: USA 54%, Canada 75%, UK 43%, France 72%, Spain 81% - Unbiased synthesis of expert opinion: USA 40%, Canada 54%, UK 50%, France 75%, Spain 46% - Will protect against patient complaints: USA 12%, Canada 32%, UK 40%, France 35%, Spain 52% - Will protect against a malpractice lawsuit: USA 10%, Canada 26%, UK 35%, France 27%, Spain 52%   **Barriers:**  Agree, strongly agree %   - Oversimplified or cookbook medicine: USA 28%, Canada 12%, UK 21%, France 7%, Spain 19% - Increase likelihood of being sued: USA 26%, Canada 7%, UK 15%, France 23%, Spain 12% - A challenge to physician authority: USA 22%, Canada 6%, UK 16%, France 13%, Spain 12% - Too rigid to apply to individual patients: USA 21%, Canada 5%, UK 16%, France 13%, Spain 12% - Too difficult to use: USA 8%, Canada 3%, UK 3%, France 6%, Spain 10% - Too time-consuming to apply: USA 8%, Canada 4%, UK 5%, France 9%, Spain 10% |  |
| Gravel (2010) | Not reported | Not reported | **Baseline characteristics of the Participants**  **Previous knowledge of the OAR (ankle aspects) on the VAS (mm), mean (95% CI)**   - Mnemonic, % (n = 95): 40.0 (23.4–46.6) - Control, % (n = 95): 42.9 (35.5–50.3)   **Previous knowledge of the OAR (foot aspects) on the VAS (mm), mean (95% CI)**   - Mnemonic, % (n = 95): 26.8 (21.2–32.4) - Control, % (n = 95): 31.3 (26.4–38.1)   **Number of perfect answers for the ankle**  **component before intervention**   - Mnemonic, % (n = 95): 0 (0.01) - Control, % (n = 95): 1 (0.01)   **Number of perfect answers for the foot**  **component before intervention**   - Mnemonic, % (n = 95): 0 - Control, % (n = 95): 2 (0.02) | **Baseline characteristics of the Participants**  **Score for the ankle component before intervention (of 13), mean (95% CI)**   - Mnemonic, % (n = 95): 3.5 (2.8–4.2) - Control, % (n = 95): 3.8 (3.0–4.6)   **Score for the foot component before intervention (of 10), mean (95% CI)**   - Mnemonic, % (n = 95): 2.3 (1.7–2.9)   Control, % (n = 95): 2.4 (1.8–3.0) |
| Hopkins (2010) |  | **Documentation of use of OAR:**  **OAR documented n=33/60:**  SHO: n=21/30 (70%)  ENP: n=12/30 (40%)  **OAR not documented n=27/60:**  SHO: n=9/30 (30%)  ENP: n=18/30 (60%) |  | **X-ray:**  Foot x-ray received: n=12/60 (20%)  Foot x-ray not received: n= 20/60 (33%)  Uncertainty of x-ray: n= 28/60 patients (47%) |
| Knox (2015) | Not reported | Not reported | **Awareness of the different types of Clinical Prediction Rules (CPR): n=211**  Ottawa Ankle Rule: n= 21/211 (10%) | Not reported |
| Lau (2013) | Not reported | **Documentation of clinical features consistent with the OAR:**  NP: n=36/51 (71%)  ED-Dr: n=101/123 (82%)   - ED Physician: n=8/9 (89%) - ED-based registrar: n=38/48 (79%) - HMO: n=47/56 (84%) - Intern: n=4/5 (80%) |  | X-ray assessment   - NP: n=40/51 (78%) - ED-Dr: n=109/123 (89%) - ED Physician: n=9/9 (100%) - ED-based registrar: n=41/48 (85%) - HMO: n=50/56 (89%) - Intern: n=4/5 (80%)   Fracture identified at ED assessment   - NP: n=9/51 (18%) - ED-Dr: n=28/123 (23%) - ED Physician: n=2/9 (22%) - ED-based registrar: n=16/48 (33%) - HMO: n=8/56 (14%) - Intern: n=0/5 (0%)   **Imaging:**   - All patients with a positive OAR feature received an X-ray - Fracture identified: n= 33/137 patients (24%) - NPs were less likely to miss a fracture during initial ED assessment compared with ED-based registrars (0% vs 29%, p = 0.013).   OAR:  No significant difference in presence of feature  NP: n= 36/51 patients (71%)  ED-Dr: n= 101/123 patients (82%)  Missed fracture at 7-day follow up   - NP: n=0/11 (0%) - ED-Dr: n=2/14 (14%) - ED Physician: n=0/9 (0%) - ED-based registrar: n=2/7 (29%) - HMO: n=0/6 (0%) - Intern: n=0/1 (0%) |
| Silveira (2016)^a^ | Not reported | **Documentation of clinical features consistent with the OAR:**  n=229/410 (56%) | Not reported | **Utilisation of radiography in the before cohorts n= 205**   - Ankle or foot: n= 198/205 (97%) - Ankle: n= 160/205 (78%) - Foot: n= 103/205 (50%)   **Fracture detected (Yield)**   - Ankle: n= 21/160 (13%) - Foot: n= 9/103 (9%) |
| Tajmir (2017)^a^ | Not reported | **Documentation of clinical features consistent with the OAR:**   - Ankle: n= 231/374 (62%) - Foot: n= 238/374 (64%) | Not reported | **Radiography use in the control group**   - Ankle only: n=124/374 (33%) - Foot only: n= 143/374 (38%) - Ankle and foot: n= 58/374 (16%) - No radiography: n= 47/374 (13%) - Total ankle: n=183/374 (49%) - Total foot: n=202/374 (54%)   **Radiography yield and fractures per patient visit of ankle and foot radiography in the control group**  All fractures   - Total: n= 35/385 (9%) - Ankle: n= 21/183 (12%) - Foot: n= 14/202 (7%)   Clinically significant fractures   - Total: n= 18/385 (5%) - Ankle: n= 9/183 (5%) - Foot: n= 9/202 (5%)   Avulsion fractures   - Total: n= 17/385 (4%) - Ankle: n= 12/183 (7%) - Foot: n= 5/202 (3%) |
| Wynn-Thomas (2002) | **GP self-reported use of ankle guidelines n=395**   - Never: n= 205/395 (52%) - Hardly ever: n=147/395 (37%) - Often: n=30/395 (8%) - Very often: n=9/395 (2%) - Always: n=4/395 (1%) |  |  | **Imaging:**  X-rays ordered for OAR positive: n= 101/110 cases (92%)  X-rays not ordered for OAR negative patients: n=58/86 (67%)  X-rays ordered for OAR negative patients: n= 28/86 cases (33%)  Implementing OAR would reduce X-ray utilisation by 16%  **OAR reliability:**  Fractures found: n= 16/133 OAR positive patients (12%)  **Usual practice reliability:**  Fractures found: n= 13/129 patients (10%) |
| **Ottawa Knee Rule** | | | | |
| Beutel (2012) | **Self-reported adherence to the Ottawa Knee Rule:**   - ED physician reported ‘never’ using the guideline: n= 17/47(36%) - ED physician reported ‘always’ or ‘most of the time’: n= 11/47 (23%) | **Documentation of clinical features consistent with OKR:**  Attending physician: n= 64/98 (66%)  Resident: n= 45/73 (62%)  Nurse: n= 35/46 (76%)  Physician assistant: n= 42/59 (71%) | **Knowledge of OKR:**   - Physicians’ mean score on was 73% 10- item knowledge questionnaire: n=47 - Respondents answering all the questions correctly: n=1/47 (2%) - Respondents answering all but 1 of the questions correctly: n=37/47 (79%) - Respondents answering all the questions incorrectly: n= 0/47 (0%)   **Barriers:**   - Patient expectation: n=31/47 (66%) - patient satisfaction: n=24 /47 (51%) - An orthopedics referral requirement: n=17/47 (36%) - Lack of confidence in physical exam findings: n=16/47 (34) - Legal concern: n=15/47 (32%) - Difficulty remembering criteria: n=13/47 (28%) - Rule is oversimplified: n=3/47 (6%) - Rule outdated or ineffective: n=1/47 (2%) - Use different clinical decision rule: n=1/47(2%) | **Radiographs ordered: n= 198/260 (76%)**   - Attending physicians: n=68/198 (34%) - Residents: n=48/198 (24%) - Nurses: n=37/198 (19%) - Physician assistants: n=45/198 (23%)   **Fracture rate:** 32/198 (16%) |
| Graham (1998) |  |  | **Physicians’ attitude towards OKR:**   - Willing to use the rule: n= 194/232 (84%) - Might use the rule: n= 32/232 (14%) |  |
| Graham (2001) | **Self-reported use of OKR**  **OKR use:**  Always/ Most of the time   - Canada: n= 63/369 (17%) - USA: n= 104/266 (39%) - UK: n= 30/297 (10%) - France: n= 16/536 (3%) - Spain: n= 11/271 (4%) | Not reported | **OKR awareness among emergency physicians:**   - Canada: n= 233/369 physicians (63%) - USA: n= 120/226 physicians (53%) - UK: n= 86/297 physicians (29%) - France: n= 66/536 physicians (12%) - Spain: n= 23/271 physicians (8%)   **Facilitators:**  Agree, strongly agree %   - Intended to cut healthcare costs: USA 85%, Canada 74%, UK 36%, France 61%, Spain 71% - Good educational tool: USA 83%, Canada 85%, UK 77%, France 77%, Spain 62% - Intended to improve quality of care: USA 79%, Canada 90%, UK 88%, France 84%, Spain 81% - A convenient source of advice: USA 79%, Canada 86%, UK 84%, France 68%, Spain 88% - Likely to decrease health care costs: USA 54%, Canada 75%, UK 43%, France 72%, Spain 81% - Unbiased synthesis of expert opinion: USA 40%, Canada 54%, UK 50%, France 75%, Spain 46% - Will protect against patient complaints: USA 12%, Canada 32%, UK 40%, France 35%, Spain 52% - Will protect against a malpractice lawsuit: USA 10%, Canada 26%, UK 35%, France 27%, Spain 52%   **Barriers:**  Agree, strongly agree %   - Oversimplified or cookbook medicine: USA 28%, Canada 12%, UK 21%, France 7%, Spain 19% - Increase likelihood of being sued: USA 26%, Canada 7%, UK 15%, France 23%, Spain 12% - A challenge to physician authority: USA 22%, Canada 6%, UK 16%, France 13%, Spain 12% - Too rigid to apply to individual patients: USA 21%, Canada 5%, UK 16%, France 13%, Spain 12% - Too difficult to use: USA 8%, Canada 3%, UK 3%, France 6%, Spain 10% - Too time-consuming to apply: USA 8%, Canada 4%, UK 5%, France 9%, Spain 10% |  |
| Knox (2015) | Not reported | Not reported | **Awareness of the different types of Clinical Prediction Rules (CPR), n=211**  - Ottawa Knee Rule: n=11/211 (5%) | Not reported |
| O’Sullivan (2006) | Not reported | **Documentation of clinical features consistent with OKR:**  **X-ray performed: n=29/43**  Fulfil OKR: n=10/29 (35%)  Did not fulfil OKR: n=19/29 (66%)  **No X-ray performed: n= 14/43**  Fulfill OKR: n= 1/14 (7%)  Did not fulfill OKR: n=13/14 (93%) | Not reported | Not reported |

CCHR= Canadian CT Head Rule, CCR= Canadian C-Spine Rule; CPR= Clinical Prediction Rules, CSR= Cervical-Spine Rule, HMO= Hospital Medical Officers, n= sample size, NP= Nurse Practitioner, ED-Dr= Emergency Department Medical Doctor, OAR= Ottawa Ankle Rules, OFAR= Ottawa Foot and Ankle Rules, OKR= Ottawa Knee Rules, VAS= Visual Analog Scale, UK: United Kingdom, US: United States

^a^ The denominator is larger than the number of patients because the denominator is the number of x-rays, occasions of services, etc.

^b^ The denominator is smaller than the number of clinicians, as the clinicians drop out of the study.
